# Mesoporous silica nanoparticles--based functional platforms for breast cancer therapy: technological advancements

**DOI:** 10.3389/fchem.2025.1741991

**Published:** 2026-01-30

**Authors:** Yang Du, Jiangnan Yang, Shuai Chen, Deyuan Fu

**Affiliations:** 1 Department of Thyroid and Breast Surgery, Clinical Medical College, Yangzhou University, Yangzhou, Jiangsu, China; 2 Department of Basic Medicine, Medical College of Hunan Normal University, Changsha, Hunan, China; 3 Department of Thyroid and Breast Surgery, Yiyang Central Hospital, Yiyang, Hunan, China; 4 Department of Thyroid and Breast Surgery, Northern Jiangsu People’s Hospital affiliated to Yangzhou University, Yangzhou, Jiangsu, China

**Keywords:** breast cancer, mesoporous silica nanoparticles, targeted drug delivery, photodynamic therapy, theranostics

## Abstract

Mesoporous silica nanoparticles (MSNs) offer remarkable opportunities for the loading and delivery of drugs and other small molecules due to their large specific surface area, tunable mesoporous pore size, ease of modification, and excellent biocompatibility. The active silica hydroxyl groups (Si-OH) on the surface provide a good basis for a variety of modification and functionalization strategies, and thus MSNs show great potential in drug delivery systems, especially in studies involving the loading, transport, and release of anticancer drugs, particularly in the field of oncology therapeutics. The current review highlights MSNs as promising platforms for breast cancer therapy, with a special focus on the diverse applications of MSNs in breast cancer therapy, which are related to a wide range of applications with different structures and designs, functionalization strategies, prevalent synthesis methods, and potential clinical uses. Afterwards, we review targeted MSNs for enhancing precision medicine and therapeutic efficacy, and then focus on specific applications of MSNs in breast cancer therapy and various stimulus-responsive surface modifications, with specific applications including drug delivery, photodynamic therapy, photothermal therapy, combined therapeutic approaches, gene therapy, and immunotherapy. In addition, the challenges of MSNs in breast cancer and their future prospects are discussed.

## 1 Introduction

The phenomenon of a younger onset of malignant tumors and their relatively high fatality rates pose a severe threat to global human health. Concerns are raised about the trend that an increasing number of the youth are being diagnosed with cancer due to changing lifestyle and environmental pollutants (Liu et al., 2023).

Moreover, cancers like breast cancer has become the most common type that is affecting women which not only has an intense effect on the quality of life but a sizable chunk of health issues for women across the globe. Over the past 3 decades global incidence of breast cancer has been increasing with an annual increase by 3.1% (Giaquinto et al., 2024). This trend is clearly observable in both developed and developing countries, with breast cancer being a leading cancer cause of mortalityin women in both areas (Bleyer and Welch, 2012). In 2018, there were an estimated 2.08 million new breast cancer cases accounting for 11.6% of all new cancer cases. What is more alarming is that the number of breast cancer–related deaths stands at 626679fatalities, accounting for about 6.6% of total deaths due to cancer (Bhattacharyya et al., 2020). The high incidence and mortality of breast cancer make it a major public health concern that requires urgent attention. Quantified by Disability-Adjusted Life Years (DALYs), which include both premature death and years of life lost due to disability, breast cancer caused approximately 20.6 million DALYs among women worldwide in 2021, a 28% increase from 1990, highlighting its long-term impact on quality of life, particularly in low- and middle-income countries. This burden is projected to exceed 30 million DALYs by 2050 without intervention (Zhu et al., 2025). From an economic perspective, breast cancer has profound societal impacts, with global costs projected to reach $21 trillion between 2020 and 2050, including direct healthcare expenditures and indirect losses due to declining productivity (Global Nutrition Target Collaborators, 2025).

Breast cancer subtypes (such as hormone receptor positive, Human Epidermal growth factor Receptor 2 (HER2) positive, and triple negative breast cancer) have an important impact on its clinical features, prognosis and therapeutic choice. Studies have shown that approximately 70% of breast cancer patients express estrogen receptor (ER)-positive, as estrogen is able to promote the proliferation of cancer cells and growth by activating ER (Hegde et al., 2021). Therefore, you can slow down the progression of the breast tumor by efficiently silencing the activity of the ER signaling pathway. Notwithstanding, Triple-negative breast cancer (TNBC) is a subtype of breast cancer that lacks expression of progesterone receptor (PR), ER, as well as HER-2 and affects about 15% of all breast cancer cases. This subtype is associated with offers the highest recurrence rate and mortality among the breast cancer subtypes with rapid progression of the disease and a high degree of heterogeneity leading to poor sensitivity to chemotherapy. TNBC usually recur early and have a higher risk of distant metastasis, which leads to a worse prognosis for patients, often associated with higher grade as well (Leon-Ferre and Goetz, 2023). As many patients remain at risk of disease recurring or advancing following treatment, it is evident that new treatment strategies as well as innovative technologies are urgently needed to combat this challenge (Hegde et al., 2023).

In this context, antitumor strategies based on functional nanomaterials have made remarkable progress in productive efficiency and clinical application. These nano-materials can not only increase the targeting and biocompatibility of drugs but also effectively reduce the toxicity to normal cells, which improves patient treatment experience and enhances efficacy. Therefore, nano-materials have played an indispensable and essential role in the development of antitumor treatment, and will open up a new direction and prospect for future work on antitumor therapies (Dai et al., 2024; Qin et al., 2024).

In recent years, the rapid development of nanotechnology has seen research efforts on various types of nanocarriers such as liposomes, polymers, and 2D nanomaterials. Despite all that, these nanocarriers have limitations, including poor drug loading capacity, less biocompatibility, and decreased biodegradability, which restrict their effectiveness in drug delivery systems. MSNs, on the other hand, as an emerging nanomaterial, have shown excellent features among these features large surface area, tunable mesoporous pore size, easily décornable surface properties, excellent biological compatibility and good bio-degradability. The above characteristics, meanwhile, give MSNs a prospect for a bright future in the load and transport of a broad range of small molecules including drugs, nucleic acids, and proteins (Mohanan et al., 2024). MSNs have exhibited excellent performance and can be an efficient means to overcome the above-mentioned limitations, which has made MSNs a very promising candidate to be applied in an area of drug delivery (Kolimi et al., 2023). Hence, MSNs nanomaterials are considered as an efficient class of nano-carrier which may offer a more reliable approach in drug delivery.

Although recent reviews of mesoporous silica nanoparticle (MSN)-based nanoplatforms for breast cancer treatment, such as those by Xu et al. (2023), Mohanan et al. (2024): Djayanti et al. (2023), Kolimi et al. (2023), have comprehensively summarized synthetic methods, functionalization strategies, and general applications, they often overlook quantitative pharmacokinetic (PK) and toxicity data that are crucial for clinical translation. For example, previous studies lacked detailed discussions of MSN-specific parameters, such as plasma half-life (>24 h after PEGylation), tumor accumulation rate (up to 10% ID/g via EPR effect), and reductions in side effects (e.g., cardiotoxicity <5%, compared to 20%–30% for free doxorubicin; hepatosplenomegaly <20%, with transient recovery within 7 days), which have been confirmed in breast cancer xenograft models and phase I clinical trials. This article reviews the multifunctional applications of MSNs in breast cancer treatment and focuses on quantitative pharmacokinetic/toxicity data and the prospects for multimodal clinical applications. This article discusses a multifunctional platform built using MSNs, where MSNs can not only serve as drug carriers but also enable targeted drug delivery and release, thereby enhancing therapeutic efficacy. Secondly, it details the research progress of MSNs as stimulus-responsive drug delivery systems, highlighting how they can regulate drug release through external stimuli (such as pH, temperature, and ultrasound) to improve the precision and timeliness of treatment. Finally, it explores the application of MSNs in image-guided tumor therapy. By combining with imaging technology, MSNs can monitor drug delivery and tumor response in real time, enabling more personalized treatment plans. Furthermore, it discusses the research on MSNs in tumor-targeting modification, particularly how to functionalize their surface to efficiently target tumor cells, thereby reducing side effects on normal tissues. Finally, it reviews the wide range of applications of MSNs in anti-tumor therapy, especially their synergistic effects in chemotherapy, gene therapy, and immunotherapy. These studies demonstrate that MSNs are valuable not only in basic treatments but also show broad application prospects in the future clinical treatment of breast cancer.

## 2 MSN overview

**FIGURE 1:**
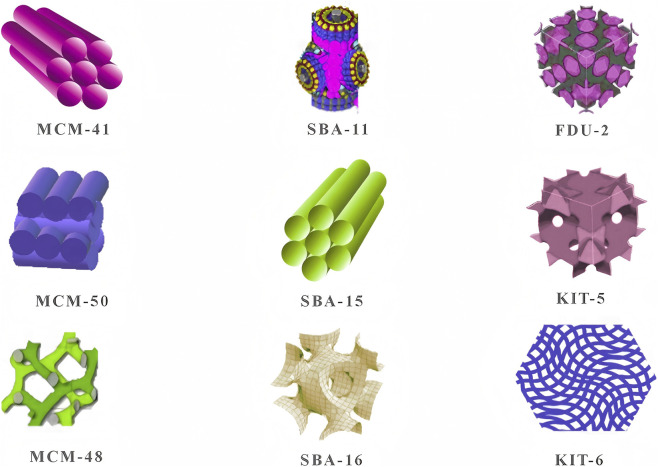
Different types of mesoporous silica nanoparticles (MSN). Reproduced from Wan and Zhao (2007).

Hierarchical mesoporous silica has a tetrahedral framework structure, consisting of one silicon atom and four oxygen atoms covalently bonded and has a regular cylindrical mesopore to be predictable pore size ranges between 2 and 50 nm (Kumar et al., 2018). Porous materials can be classified into three types according to the pore size: microporous, mesoporous, and macroporous. Where the pore sizes of micropores are typically less than 2 nm, mesopores have pores between 2 and 50 nm, and macropores have pores greater than 50 nm. Among the various porous materials, mesoporous materials are particularly important, including Mobil Composition of Matter (MCM), Santa Barbara Amorphous (SBA), Korea Advanced Institute of Science and Technology (KIT), and Fudan University (FDU) series (Narayan et al., 2018), as shown in Figure 1; Table 1. These materials have shown excellent performance in catalysis, gas adsorption, and drug delivery and are consequently widely used. Different types of mesoporous silica materials (MSN) possess diverse mesoporous structures, pore channels, and pore sizes, hence it is possible to choose the right material according to the properties it will require for specific applications (Li and Zhou, 2023). MCM-41, MCM-48 and MCM-50 are mesoporous Silica materials with introduction channels, however each has various paths for introducing the synthesis and application As there are a number of issues like instability and other limitations to their mesoporous structure, the three systems are considered unreliable or undesirable for many materials (Kumar et al., 2017). Because of their huge surface areas and adjustable pore sizes, the MCM series had an important role in the development of molecular sieves. MCM-41 was the first meso-structured material successfully synthesized and their subsequent generations of MCM-48 and MCM-50 showed clear benefits of their synthesis as these materials reveal a vast variability and superiority that make indispensable in the mesoporous materials research area, especially the MCM-41, this material has not only been a significant milestone of ordered mesoporous material but also exhibited numerous potential applications.

**TABLE 1:** Characterization of nanocarriers for different classes of MSNs.

MSNs	Syngony	Silica source	Pore diameter (nm)	References
MCM-41	2D hexagonal	TEOS	2.0–5.0	Wu and Bein (1996)
MCM-50	3D cubic	TEOS	2.0–5.0	Li and Zhou (2023)
MCM-48	Lamellar	TEOS	2.0–5.0	Li et al. (2019)
SBA-11	3D cubic	TEOS	5.0–8.0	Narayan et al. (2018)
SBA-15	2D hexagonal	TEOS	5.0–10.0	Xu et al. (2023)
SBA-16	3D cubic cages	TEOS	1.0–9.0	Kumar et al. (2017)
FDU-2	3D cubic	TEOS	4.0–10.0	Jafari et al. (2019)
KIT-5	3D cage-like	TEOS	5.0–10.0	Zhu et al. (2017)
KIT-6	3D bicontinuous cubic	TEOS	4.0–12.0	Moodley and Singh (2021)

**FIGURE 2:**
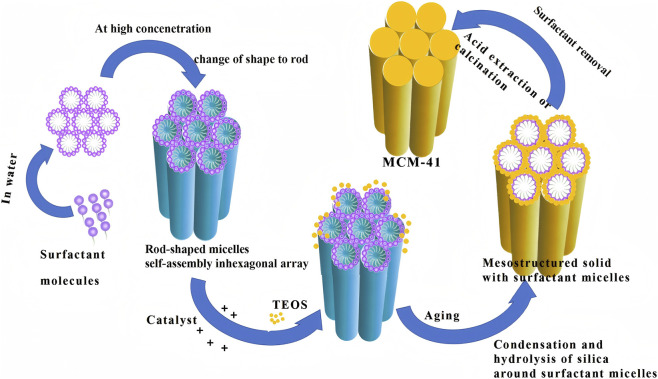
Synthesis schematic of MCM-41 (This figure was drawn by Figdraw).

**FIGURE 3:**
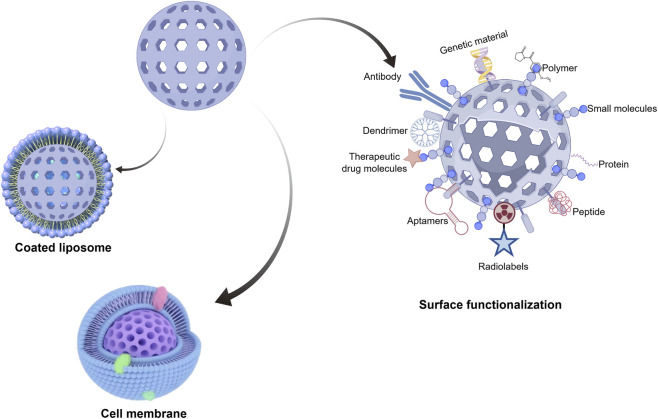
Surface functionalization of the MSNs in breast cancer treatment (This figure was drawn by Figdraw).

Although various types of MSNs (MCM, SBA, KIT, FDU series, etc.) have been developed, their practical application in breast cancer therapy is ultimately determined by controllable synthesis and systematic surface/channel functionalization. The most widely adopted synthesis strategies and corresponding key physicochemical properties critical for tumor targeting, drug loading, and stimuli-responsive release are summarized in Figure 2; Table 2, while the three major functionalization approaches currently used to confer tumor-specific recognition and intelligent delivery functions are illustrated in Figure 3; Table 3.

**TABLE 2:** Synthesis methods of mesoporous silica nanoparticles (MSNs) and characteristics for breast cancer therapy.

Synthesis method	Core principle and key steps	Key tunable parameters	Resulting MSN properties critical for breast cancer therapy	Advantages, limitations and specific applications in breast cancer	Representative references
Sol-Gel Method	Surfactant (e.g., CTAB) forms rod-like micelles in water → micelles self-assemble into hexagonal arrays at high concentration → addition of TEOS + catalyst → hydrolysis and condensation of silica around micelles → aging → template removal by calcination/extraction (complete MCM-41 process shown in Figure 2)	pH, silicon source concentration and addition rate, TEA addition, co-condensation of phosphonate/carboxylate groups	Particle size 50–150 nm, PDI <0.10, zeta ≤−45 mV, high monodispersity, stable >7 days in 10% FBS; significantly enhances EPR effect and deep tumor penetration, reduces liver/spleen non-specific uptake	Currently the dominant method for targeted delivery in breast cancer; anti-aggregation optimization has become the gold standard for clinical translation	Nakao et al. (2012), Kolimi et al. (2023), Kumar et al. (2017), Kumar et al. (2018), Li et al. (2019), Li and Zhou (2023), Narayan et al. (2018)
Hydrothermal Synthesis	Surfactant templating → pH adjustment with acid/base → addition of silica source → crystallization in autoclave under high temperature/pressure → washing and calcination to remove template	Temperature (100 °C–200 °C), reaction time, acid/base type, surfactant selection	Highly tunable pore size and morphology (spherical, rod-like, hollow, etc.); drug loading >20% w/w; release >70% in acidic TME; tumor inhibition >80% in 4T1 models	Ideal for high drug-loading and multi-drug co-delivery systems, especially in triple-negative breast cancer (TNBC)	Kumar et al. (2017), Mohanan et al. (2024)
Microwave-Assisted Synthesis	Rapid and uniform heating via dielectric polarization of polar molecules (water/alcohols), dramatically shortening reaction time to minutes	Microwave frequency (915–2450 MHz), power, solvent polarity	Uniform pore channels, high synthesis efficiency; facile preparation of stimuli-responsive MSNs; >65% PDT-induced cell death in MDA-MB-231 cells	Fast, green, and scalable; particularly suitable for PDT/SDT synergistic platforms	Wu and Bein (1996)

**TABLE 3:** Functionalization strategies of mesoporous silica nanoparticles (MSNs) and applications in breast cancer therapy.

Functionalization type	Description	Methods/Examples	Benefits/Applications in breast cancer therapy	Representative references
Surface Functionalization	Covalent or physical attachment of organic molecules, polymers, or inorganic materials to MSN surfaces via abundant silanol groups (Si-OH)	Functional groups: carboxyl, amino, thiol Polymers: (3-aminopropyl)triethoxysilane, polylysine, polyethyleneimine Hydrophobic modification: mixtures of bis(2-methoxyethyl)dimethylsilane, chlorotrimethylsilane, poly (methylhydrosiloxane) Image examples: Antibody, Polymer, Small molecules, Protein, Peptide, Aptamers, Radiolabels	Enhances biomolecule interactions, improves drug loading and controlled release, significantly increases loading efficiency of hydrophobic drugs; enables better biocompatibility and targeted delivery in breast cancer	Bartusik-Aebisher et al. (2025), Manzano and Vallet-Regí (2020), Wang et al. (2015), Wei et al. (2024)
Portal (Pore Channel) Functionalization	Chemical modification inside mesoporous channels to confer responsiveness to environmental stimuli (pH, temperature, light, magnetic field)	Incorporation of pH-responsive polymers, metal nanoparticles, or enzymes inside pores Image examples: Genetic material, Dendrimer, Therapeutic drug molecules	Achieves stimuli-responsive and triggered drug release (especially in acidic TME), enhances catalytic activity, enables precise therapy and probe design in tumor microenvironment	Braga et al. (2024), Chakraborty et al. (2025), Kamil Mohammad Al-Mosawi et al. (2023), Liu et al. (2024), Patel et al. (2025), Rahman et al. (2023), Zheng et al. (2018), Zhuo et al. (2023)
Surface Coating and Gatekeeper Functionalization	External coating with functional layers or installation of molecular “gatekeeper” systems on MSN surface	Coatings: liposomes, polymers, tumor cell membranes Functional nanoparticles: gold NPs, magnetic NPs Molecular gatekeepers Image examples: Coated liposome, Cell membrane	Dramatically improves biocompatibility, *in vivo* stability, and tumor targeting; realizes “smart switch” control; particularly suitable for multimodal synergistic therapy (chemo-PTT-PDT-immunotherapy) in breast cancer	Chen et al. (2024a), Croissant et al. (2016), Tang et al. (2024a), Xu et al. (2023)

## 3 MSN in anti-tumor applications

Currently, the most widely used metal-like nanomaterials in the biomedical field include silica nanoparticles (SiO2NPs), carbon nanomaterials (e.g., graphene, fullerenes, carbon nanotubes), and black phosphorus nanosheets (BP Nanosheets), and silica nanoparticles have shown many advantages in the application of tumor therapy due to their unique properties (Manzano And Vallet-Regí, 2020).

In terms of biosafety, most experiments have shown that silica nanoparticles are biocompatible, and their slow degradation product, protosilicic acid [Si(OH)4], is less toxic and can be excreted via the excretory system. Second, SiO_2_ NPs have high drug-loading capacity, easy surface modification, and low toxicity, and their porous structure improves the loading efficiency of drugs and molecules. Therefore it is easier to design and construct anti-tumor nanoplatforms based on silica nanoparticles. In addition to these advantages, SiO_2_ NPs have comparable or even better performance in tumor therapy than other metal-like materials, such as multimodal synergistic therapies developed based on MSNs, multiple imaging capabilities, and good prospects for applications in biocatalysis (Wang et al., 2015).

Recently, related researchers developed a pH-sensitive, targeted aspirin (Asp) nano-delivery system for controlled release and targeted therapy of hepatocellular carcinoma by preparing Gal-modified Polydopamine (PDA)-coated mesoporous silica nanoparticles (Gal-PDA-MSN). It was demonstrated that the system has therapeutic potential by accelerating drug release in acidic environment and inhibiting HepG2 cells better than free Asp (Wei et al., 2024).

Tang et al. (2024a) develope that the SP94 modified lipid bilayer-coated mesoporous silica nanoparticles (SP94-LB@BA-MSN) as a tumor-targeting boron delivery agent for the boron neutron capture therapy (BNCT) of liver cell carcinoma. The nanocarrier has been proved to be effective in preventing premature release of the drug, in enhancing tumor-selective targeting, and presenting excellent activity in cell and mouse models. This study told us that the content of boron in the tumor tissue was distinctly higher than that in normal tissue and blood, which enlightens the tremendous potential of the material for BNCT therapy and underscores the advantages of the mesoporous silica nanoparticles. Liu et al. (2024) reported SDC1-modified lipid bilayer-coated mesoporous silica nanoparticles (SDC1-LB-MSN) for targeted delivery of both gemcitabine (GEM) and honokiol (HNK) in treating pancreatic cancer. This nanocarrier system killed selectively tumor cells and *in vivo* animals finding showed a substantial apoptosis índex completely superior to that sown by single drugs or unmodified nanoparticles. This strategy enhances the therapeutic effect of pancreatic cancer by simultaneous targeting of tumor and stroma cells.

For the multimodal treatment of malignant tumors, Zhuo’s latest research developed a nanocomposite (SiO2@CaO2@DOX@P53-HA) that achieves high drug loading through a mesoporous silica framework and incorporates hyaluronic acid to improve targeting. The material significantly enhanced tumor drug enrichment and pro-apoptotic effects, and effectively inhibited tumor growth while protecting normal tissues in a mouse model. The study suggests that this multimodal nanotherapeutic has great potential to improve the therapeutic efficacy of lung cancer treatment (Zhuo et al., 2023). Kamil Mohammad Al-Mosawi et al. (2023) has developed a magnetic mesoporous silica nanoparticle with imaging and therapeutic functionality for targeted therapy and diagnosis of colorectal cancer. The particles were loaded with 5-fluorouracil (5-FU) drug, surface-modified with polyethylene glycol (PEG) and Epithelial cell adhesion molecule (EpCAM) aptamers for pH-dependent controlled release via Au nanoparticles. Experiments showed the carrier continuously released the drug under acidic environment with high uptake efficiency and significant anticancer effect on cancer cells, and *in vivo* studies verified that it effectively inhibited tumor growth with fewer side effects, including reduced cardiotoxicity (incidence <5% vs. 20%–30% in free DOX) and nephrotoxicity (<10% vs. 15%–25% in conventional chemotherapy) in mouse models of colorectal cancer. Despite the excellent safety profile of MSNs in anti-tumor applications, potential side effects still warrant attention, primarily including transient inflammation due to hepatosplenic accumulation, mild nephrotoxicity due to delayed renal clearance, and potential immunogenicity when the surface is not optimized. *In vivo* studies in breast cancer have shown that the incidence of these side effects is significantly lower than that of conventional chemotherapy: ① Cardiotoxicity: After loading DOX onto MSNs, the incidence of myocardial injury decreased to <5% (equivalent to 0%–3% of patients at human doses in mouse models), compared to 20%–30% with free DOX (Braga et al., 2024; Chakraborty et al., 2025; Patel et al., 2025). Nephrotoxicity: Serum creatinine elevation <10% (4T1 breast cancer mouse model, n = 20), compared to 15%–25% with conventional nanomedicines (such as Abraxane®) (Zheng et al., 2018). ② Hepatosplenic loss: alanine aminotransferase (ALT)/aspartate aminotransferase (AST) elevation of 5%–15% (transient, recovering in <7 days), occurring in <20% of test animals; in the clinical Phase I trial (n = 16, prostate cancer patients, similar to MSNs), only 6% of patients reported mild liver enzyme abnormalities.” (Rahman et al., 2023). ③ Immune-related adverse events (irAEs): In multimodal MSNs (such as those combined with programmed death 1 (PD-1) inhibitors), the incidence of cytokine storms was <5% (TNBC mouse model), far lower than the 10%–20% of immune checkpoint inhibitor (ICI) monotherapy (Chen Y. et al., 2024). These quantitative data are derived from breast cancer xenograft models (n = 10–30/group) and limited clinical trials, showing that MSNs significantly reduce systemic toxicity (overall adverse event rate <15% vs. 40%–60% of chemotherapy) through enhanced permeation and retention (EPR) effect and surface PEGylation. Future large-scale Phase III trials will further validate these advantages.

**FIGURE 4:**
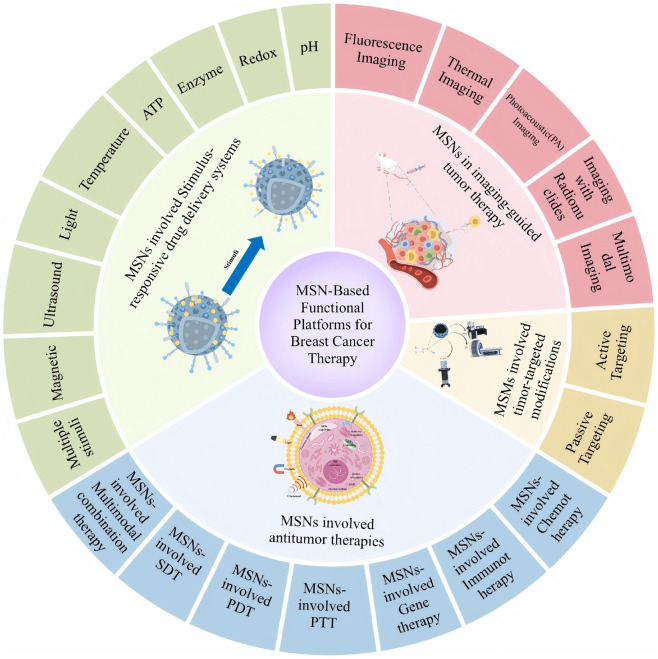
Schematic illustration of the use of MSNs in breast cancer treatment.

Figure 4 graphically illustrates the multifunctional application of monodisperse silica (MSNs) in breast cancer therapy. The following key elements are included in the figure: ① MSNs stimulus-responsive drug delivery system: the figure shows how MSNs can trigger drug release through external stimuli (e.g., pH, temperature, light, or ultrasound) to achieve precise and controlled treatment. ② MSNs in imaging-guided tumor therapy: by combining imaging technologies (e.g., fluorescence imaging, MRI, etc.), MSNs can monitor the drug delivery process and therapeutic response in real time to further enhance the precision of treatment. ③ Tumor-targeting modification of MSNs: the illustration shows the surface functionalization of MSNs, such as binding specific ligands or antibodies, to enhance their targeting of breast cancer cells and reduce the toxic side effects on normal cells. ④ Role of MSNs in anti-tumor therapy: MSNs can not only be used in chemotherapy, but also combined with other therapeutic modalities, such as immunotherapy, gene therapy, photothermal, photodynamic, and acoustic therapy, to enhance therapeutic effects. This figure comprehensively demonstrates the wide application of MSNs in breast cancer treatment and their potential for multimodal combination therapy, highlighting their advantages in targeting, drug release control and therapeutic synergy.

## 4 Surface modification of MSNs nanomaterials for breast cancer therapy

**FIGURE 5:**
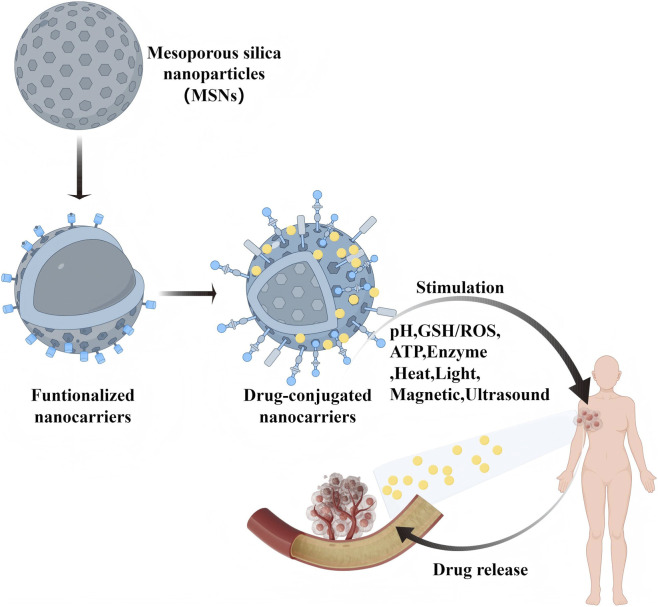
Flowchart of drug delivery system drug delivery for MSNs (This figure was drawn by Figdraw).

Unsurface-modified porous silica materials (MSNs) suffer from many functional limitations, while their drug loading capacity as well as the modulation of drug release rate are significantly reduced. Therefore, rational surface modification of MSNs for targeted drug delivery and responsive release has become an important hotspot in today’s research. In recent years, numerous researchers have successfully developed a variety of MSNs with environmental stimulus-responsive properties, and these materials are capable of intelligent drug release in response to environmental stimuli. In the existing environmental stimulus responsive studies, the carriers prepared by using endogenous signals such as pH, redox reactions, enzyme activities and adenosine triphosphate, which are specific to diseases such as tumors, are called endogenous stimulus responsive MSNs carriers, while the carriers made by applying exogenous signals at the lesion site, such as temperature, magnetic field, light and ultrasonic waves, are called exogenous stimulus-responsive MSNs carriers (Zhao et al., 2024). After surface functionalization, the hydrodynamic diameter of mesoporous silica nanoparticles typically increases by 10–50 nm. The hydrodynamic diameter of bare MSNs is generally 80–150 nm, while after surface functionalization, it typically increases to 100–200 nm (Chen et al., 2025). This increase in diameter mainly comes from the physical thickness of the surface modification layer (such as silane coupling agents, polymers, etc.) and the contribution of the hydration layer around the modification layer. The change in hydrodynamic diameter caused by surface functionalization has a dual impact on the role of MSNs as drug delivery carriers. When the modified hydrodynamic diameter is maintained below 200 nm, this moderate increase in diameter is usually beneficial because the surface modification layer can significantly reduce the adsorption of plasma proteins, thereby prolonging the circulation time of MSNs in the blood and improving the passive targeting effect of tumors. At the same time, the moderate increase in hydrodynamic diameter does not significantly impair the EPR effect. Instead, it effectively reduces the aggregation of modified MSNs in the blood circulation by improving colloidal stability, thereby increasing the number of effective particles that actually reach the tumor site. However, when surface modification results in a hydrodynamic diameter that significantly exceeds 250–300 nm, it has a negative impact on drug delivery efficiency, mainly manifested as a significant decrease in tumor stroma penetration and an increase in clearance by the mononuclear phagocytic system (Djayanti et al., 2023). As shown in Figure 5 is the Flowchart of drug delivery system drug delivery for MSNs.

## 5 Stimulus responsiveness modification

**FIGURE 6:**
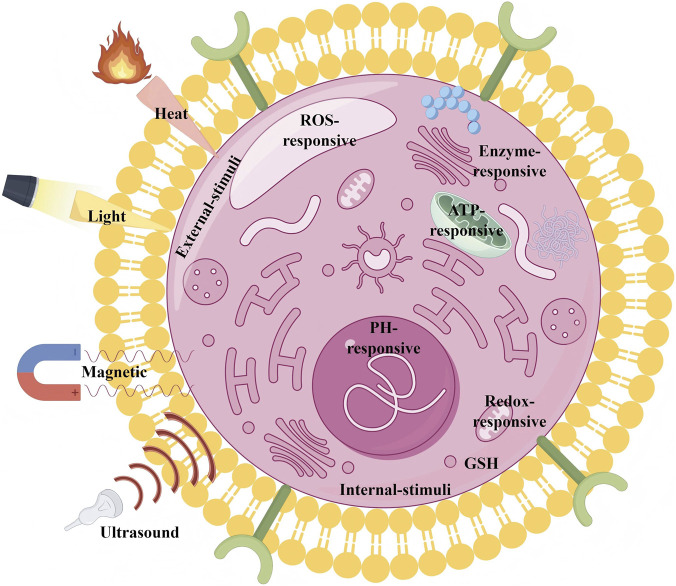
Schematic representation of different types of stimulus-responsive MSNs drug delivery systems (This figure was drawn by Figdraw).

Surface functionalization of MSNs is one of the key strategies to enhance their therapeutic efficacy and precision, with stimulus-responsive modifications being particularly important. These modifications enable MSNs to trigger drug release in response to specific external or endogenous stimuli for precise targeting and controlled therapy. Common stimulus-responsive functions include responses to pH, redox state, enzymes, adenosine triphosphate (ATP), temperature, light, ultrasound, and magnetic fields, in addition to multiple stimulus responses to adapt to the complex demands of the tumor microenvironment. In this paper, we will briefly introduce the stimulus-responsive modifications on the surface of MSNs and their promising applications in precision drug delivery, as detailed in Table 4; Figure 6.

**TABLE 4:** Common responsive MSNs nano-delivery systems and features in the treatment of breast cancer.

Responsive drug delivery systems	Stimulus-responsive categories	Mechanisms of action	Nanocarrier	Drug loading	Cell lineage	Advantages	Limitations	References
pH-responsive	pH	The extracellular pH of malignant tissues is usually in the slightly acidic range of about 6.0–7.0. this contrasts markedly with the normal pH of 7.4 in healthy tissues. When the pH in the environment drops below a certain threshold, the surface charge of the material drug-carrying systems (MSNs) is flipped, along with the breaking of chemical bonds. This process activates the drug carriers, allowing them to release the drug efficiently, thus ensuring effective drug release	Chr- mSiO2@PAA/FA	Chrysin	MCF-7	A significant gradient exists between the acidic tumor microenvironment (pH 5.0–6.8) and normal tissue (pH 7.4); the response speed is rapid; and the response range is well-defined	The small pH gradient inside tumors may lead to incomplete drug release; some polymers may degrade prematurely in the acidic environment outside the tumor, affecting drug utilization	Ghosh et al. (2023), Nasri et al. (2023), Ramezani-Aliakbari et al. (2022), Zhang et al. (2021a)
Redox-responsive	GSH/ROS	There is a difference in the redox state in tumors and healthy tissues, and this difference allows GSH levels to vary in the microenvironment. This change can induce disulfide or diselenide bond breakage in GSH-sensitive MSNs, thereby effectively facilitating drug delivery at the tumor site	MSN-NH2@PTX@SiSS-PEG	PTX	MCF-7	There is a significant difference in GSH concentration between intracellular (2–10 mM) and extracellular (2–20 μM) tumor cells; the response is highly specific	GSH concentrations vary considerably among different tumor types and individuals; prolonged treatment may lead to intracellular GSH depletion, affecting response efficiency	Liu et al. (2020a), Zhu et al. (2021)
Enzyme-responsive	Enzyme	Based on the properties of enzyme-catalyzed reactions, upregulated enzymes can act as triggers for stimuli-responsive porous silica nanoparticles (MSNs) under different pathological conditions, thereby effectively promoting multiple chemical reactions in cancer tissues	MSN-YSA-DOX	DOX	SKBR3, MDA-MB-231, MCF7	Multiple proteases (such as MMPs and uPA) are highly expressed in the tumor microenvironment; enzyme catalysis exhibits an amplification effect; and the response is highly specific	Enzyme concentration and activity exhibit significant spatial heterogeneity within tumors; enzyme expression levels may vary depending on tumor type and stage; enzyme mutations may lead to response failure	Peng et al. (2024), Shahbaz et al. (2024), Zhang et al. (2023a)
ATP-responsive	ATP	The proliferative metabolic activity of tumor tissues was significantly higher than that of normal tissues, and the activity of mitochondria was more vigorous, resulting in the expression level of ATP reaching 1.2 times that of normal tissues. Based on this difference in ATP expression levels at the site of pathogenesis, we designed porous silica nanoparticles (MSNs) with ATP-sensitive materials modified on the surface, thus constructing a drug release system that responds to ATP stimulation and realizes effective drug delivery	MP-SiO2 NPs	CPT	MDA-231, MCF-10a	The intracellular ATP concentration (1–10 mM) in hypermetabolic tumor cells such as triple-negative breast cancer is significantly higher than that in normal cells; the response exhibits good selectivity	ATP concentration exhibits a gradient within the tumor; ATPase activity may interfere with the response process; ATP levels vary considerably across different tumor types and treatment stages	Jiang et al. (2022)
Temperature-responsive	Temperature	The temperature of tumor or inflammation sites is usually 4–5 °C higher than that of normal tissues. By taking advantage of the temperature difference at the site of pathogenesis, we have designed surface-modified temperature-sensitive materials for MSNs to construct drug-release systems with temperature-stimulation-responsive properties, thereby exploiting the temperature difference to achieve drug delivery	Au@MSN-PTX@CPT	PTX, CPT	4T1	The local high temperature of the tumor (39 °C–42 °C) can be used as an endogenous trigger signal; precise spatiotemporal control can be achieved through exogenous heating	Temperature sensitivity may cause drugs to be released prematurely before reaching the tumor; precise temperature control is required to avoid thermal damage to surrounding normal tissues	Zhong et al. (2022), Zhou et al. (2022)
Photo-response	Light	The introduction of light-triggered systems in MSNs makes photodynamic (PDT) or photothermal therapy (PTT) a viable treatment modality	CuS@mSiO2-Pc (DOX)@HA	DOX	4T1	It exhibits excellent spatiotemporal selectivity, enabling precise control of the location and timing of drug release; it also allows for non-contact regulation	Light has limited penetration depth into tissues (near-infrared light approximately 1–3 cm); it may cause phototoxicity; and the control of light conditions requires high precision	Li et al. (2024a), Lv et al. (2022), Vankayala and Hwang (2018), Wang et al. (2020), Yang et al. (2022)
Ultrasonic-responsive	Ultrasound	MSNs-based systems can be used for ultrasound triggering to effectively deliver drugs to cancer sites	MSN-DOX-Ce6	DOX, Ce6	MDA-MB-231	Ultrasound has a large penetration depth, enabling treatment of deep tissues; it is non-invasive; and it allows for a large treatment area	Optimizing ultrasound parameters (frequency, intensity, duration) is complex; cavitation effects may cause mechanical damage to surrounding tissues; and the equipment requirements are high	Manzano and Vallet-Regí (2019), Tehrani Fateh et al. (2021), Xu et al. (2020)
Magnetic-responsive	Magnetic field	Developing Magnetic MSNs to Carry Tumor Therapeutics by Binding Magnetic Particles to Drug Molecules and Applying External Magnetic Fields to Tumor Sites to Activate Drug Release	CUR/SIL mSiO2@SPIONs	CUR, SIL	MCF-7	Precise position control can be achieved through an external magnetic field; no tissue penetration is required; rapid response	Magnetic field strength decreases inversely cubically with distance, making it difficult to control deep tissues; magnetic nanoparticles may generate thermal and spin effects	Ghosal et al. (2022), Laranjeira et al. (2022), Portilho et al. (2018), Romaní -Cubells et al. (2024), Sadegha et al. (2022), Yao et al. (2017)
Multiple stimulus-responsive	Multiple Stimulating Factors	Combined effects of multiple stimuli	DOX@HMSN–SS-PLL	DOX	HeLa, MCF-7, 4T1	The synergistic effect of multiple stimuli can improve response specificity and reliability; it can also lower the response threshold of a single stimulus and improve response efficiency	The complexity of design and preparation increases significantly; interactions between different stimuli may lead to unpredictable response behaviors; and precise coordination of multiple stimulus conditions is required	Chen et al. (2020), Moodley and Singh (2021), Zhao et al. (2024)

### 5.1 pH-responsive MSNs

PDA coating not only effectively covers drug-loaded MSNs nanoparticles but also presents remarkable regulatory ability in drug release. This regulation mechanism is particularly important for anticancer drugs, since controlled release of the medicine is believed to be an effective way to improve the therapeutic effect of cancer. Additionally, the pH sensitivity of PDA coating is another advantage, especially in acidic environments, PDA will automatically dissociate from the surface and release the encapsulated drugs. This feature is of great practical significance for the treatment of targets on cancer cells. Ramezani has developed targeted PDA coated MSNs for pH-sensitive delivery of manganese molybdenum acid for the treatment of breast cancer. PDA-MSN-Glu showed the highest drug release rate shifting in acidic environment (i.e., pH 5.0) and a lower release rate in neutral environment (pH 7.4) which will make it better release from the nanoparticles in acidic environments such as inflammation or cancer and consequently enhance therapeutic outcomes (Ramezani-Aliakbari et al., 2022).

Zhang et al. (2021a) prepared a mesoporous silica nanoparticle coated with polyacrylic acid (PAA) and pH-sensitive lipid (PSL) for the delivery of arsenic trioxide (ATO) and paclitaxel (PTX), and the drug-carrying nanoparticles showed dual pH responsiveness (pHE = 6.5, pHendo = 5.0) and sequential drug release profile. In PSL, PTX was preferentially released at pH = 6.5, whereas ATO was mainly released at pH = 5.0, achieving dual pH responsiveness triggering sequential drug release under different acidic conditions, which demonstrated a longer cycle time, significant tumor-targeting delivery ability, and overall therapeutic efficacy enhancement. Nasri et al. (2023) prepared a 1,2-distearoyl-sn-glycero-3-phosphoethanolamine-citric anhydride (DSPE) nanoparticle. -polyethylene glycol (DSPE-CA-PEG) polymer, a pH- and heat-sensitive lipid-coated mesoporous silica nanoparticle (L-MSN), which triggered drug release in the acidic microenvironment (pH ≈ 5) and high temperature (42 °C) conditions of cancer cells for efficient synergistic therapy. Ghosh et al. (2023) used mesoporous silica nanoparticles (Chr-mSiO2@PAA/FA) were folic acid conjugated polyacrylic acid capped with pH responsive properties. These nanoparticles release salicin in acidic environments (e.g., low pH of the tumor microenvironment), which enhances anticancer efficacy. Through this pH-dependent targeted delivery mechanism, salicin is able to induce apoptosis in MCF-7 cells through oxidative damage and mitochondrial dysfunction and lead to G1 phase arrest of the cell cycle. Lee et al. (2022) have developed a mesoporous silica nanoparticle coated with mannose grafted polyacrylic acid copolymer (DOX@MSNs-man-g-PAA) by developing a nanosystem that shows pH-dependent drug release, excellent hemocompatibility, and significant enhancement of drug uptake by breast cancer cells through mannose receptor-mediated cytophagy, improving the hemocompatibility and cancer cell targeting ability of conventional MSNs.

### 5.2 Redox-responsive MSNs

Glutathione (GSH) is an important endogenous reducing agent with low concentrations in blood and extracellular fluids and significantly higher concentrations in normal cells. Remarkably, in tumor cells, the concentration of GSH is strikingly four times that of normal cells. This phenomenon is suggestive of the unique biochemical environment of tumor cells (Zhu et al., 2021). In addition, the concentration of hydrogen peroxide (H2O2) was also significantly higher in tumor cells than in normal cells, which adds an important dimension to the physiological properties of tumor cells. In response to this difference in GSH and H2O2 concentrations in tumor cells, researchers have innovatively developed polymers and porous materials (e.g., porous silica nanoparticles, MSNs) containing reduction- and oxidation-sensitive groups, aiming at a redox-responsive controlled-release mechanism of drugs (Liu J. T. et al., 2020).

The use of disulfide or diselenide bonds to link drug molecules is able to significantly reduce the toxicity of a drug while increasing its potency. In this way, drug release interacts with the intracellular redox state, leading to specific targeting of tumor cells. In summary, regulating intracellular redox homeostasis is not only an important component of understanding tumor biology, but also a key approach to breast cancer growth. By regulating the intracellular environment, it will be able to provide new ideas and strategies for the treatment of breast cancer (Sarmiento-Salinas et al., 2019).

Zhang et al. designed a unique hollow mesoporous silica nanoparticles (HMSN) encapsulated with folic acid-modified bovine serum albumin (BSA-FA) for tumor targeting and dual pH/redox responsive drug release of loaded methylene blue and adriamycin. The drug release mechanism was dependent on the pH and redox environment and was achieved through imine bonding between HMSN-CHO and BSA-FA and disulfide bond cleavage within BSA (Zhang et al., 2023a). Shahbaz et al. also constructed a redox-responsive drug delivery system with a core of MSNs loaded with PTX and functionalized with disulfide bonds through a nonporous silica shells and PEG encapsulation, exhibited redox-responsive drug release properties at different GSH concentrations, significantly improved cellular uptake efficiency, and induced apoptosis, demonstrating potential applications in breast cancer therapy (Shahbaz et al., 2024). Peng and other related researchers developed a dual-sensitive lipid composite nanoparticle (HMSN-OXP-Lip), HMSN-OXP-Lip depletes intracellular GSH and inhibits its production by reducing disulfide bonds in HMSNs and Lip cleavage, leading to elevated levels of reactive oxygen species (ROS), which induces immunogenic cell death (ICD) effects and activates immune responses, while delivering the chemotherapeutic agent oxaliplatin (OXP), which enhances the therapeutic efficacy of breast cancer treatment (Peng et al., 2024).

In response to tumor microenvironment (TME) multifunctional smart nanodelivery system for targeting metastatic breast cancer with combined iron death and chemotherapy, Nourollahian et al. developed an Apt-PEG-Silica-DOT@DOX multifunctional nanoparticle, which was delivered by loading adriamycin (DOX), as well as AS1411 aptamer aptamer modification to enhance tumor targeting, and the system demonstrated significant anti-tumor effects in 4T1 breast cancer cells and mouse models, with tumor inhibition rates as high as 90% (Nourollahian et al., 2024). Jiang et al. similarly developed a gold-doped mesoporous silica nanoparticles (Au-MSNs), which is a redox-responsive mesoporous silica nanodrug delivery system. In this study, Au-MSNs enhanced the redox response of the cell membrane by promoting the generation of ROS, thereby improving cell permeability and therapeutic efficacy (Jiang et al., 2022).

### 5.3 Enzyme-responsive MSNs

Endogenous enzymatic reactions in changing the TME is key. It gives new ideas for targeted drug delivery and controlled release. In an inflammatory TME, many enzymes like proteases, phosphatases, kinases, and oxidoreductases are overly secreted. This not only affects tumor growth and spread but also opens doors for designing drug delivery systems. Studies show that natural enzymes have unusual activity, substrate specificity, and strong catalytic ability in many cancers. These traits offer unique benefits. They allow us to design drug systems aimed at certain enzyme types. Key design points include linking enzyme substrates to nanocarriers and causing bonds to form or break during reactions. Such strategies make it possible to use enzyme specificity and catalytic power to boost accuracy and results in cancer treatments (Xiong et al., 2024).

Enzyme responsive systems, for specificity, sensitivity and catalytic action are full of potential for translation to biomedicine, particularly for tumor microenvironment remodeling. Not only they enable the identification of specific targets with high precision, but also stimulate biological responses under specific circumstances and actively remold the tumor microenvironment, finally improving therapeutic outcomes. Yet, the catalytic effects of the enzyme stimulated systems may have strict requirements in terms of environmental conditions, such as temperature, pH, etc. Due to the effects of EPR effects, the distribution of drugs within the tumor tissue is often uneven, leading to a reduction in therapeutic efficacy (Edwards et al., 2021). Moreover, current strategies for passively targeting the tumor microenvironment often face the problems of poor drug delivery efficiency and insufficient stability (Wang D. et al., 2019; Zeng et al., 2021).

Liu et al. developed a new MSN-YSA-DOX nanoparticle for EphA2-targeted delivery to breast cancer cells while loaded with the chemotherapeutic agent doxorubicin. Eph receptors are the largest family of receptor tyrosine kinases and are classified into types A and B. Eph receptors play a critical role in embryonic development and human diseases, including cancer. Diseases, including cancer. EphA2 is expressed in breast cancer cells and plays a role in breast cancer initiation, progression, and prognosis. MSN-YSA-DOX nanoparticles were used to enhance delivery of therapeutic agents to EphA2-expressing breast cancer cells and potentially reduce toxicity while enhancing the therapeutic efficacy of the breast cancer treatment (Liu et al., 2018).

In addition to traditional enzyme-responsive MSNs, protein-based multiaptamer recognition has shown great potential as an efficient alternative strategy. Multiaptamers can significantly enhance the cellular uptake efficiency of MSNs by simultaneously targeting multiple protein markers (such as HER2 and EphA2) on the surface of tumor cells, achieving high affinity and specific binding. In the field of breast cancer, Wang et al. (2024) developed a detection system based on multiaptamer-functionalized MSNs. This system loads different dyes (hematoxylin or curcumin) as signal transducers, which work synergistically with aptamer-modified magnetic beads to achieve colorimetric detection of MCF-7 cells (linear range 100–4000 cells/mL, detection limit 10 cells/mL), and has been successfully applied to the diagnosis of breast cancer patients in real blood samples (specificity >95%). This strategy can be further integrated with enzyme-responsive gating, for example, by using proteases (such as MMPs) in the tumor microenvironment to degrade the multiaptamer layer to trigger drug release, thereby realizing an integrated platform of “recognition-response-delivery”. Compared to single-enzyme responses, the multi-aptamer approach is more robust in complex tumor heterogeneous environments, but requires optimization of MSN surface density to avoid steric hindrance. This method provides a new paradigm for precision diagnosis and treatment of breast cancer and can be extended to multimodal therapies in the future (Chou et al., 2021).

### 5.4 ATP-responsive MSNs

ATP is a critical metabolic product in humans and its level will vary with the changes in a person’s physiological status (Mo et al., 2014). Among the various disease states, TNBC is characterized by high proliferative metabolism, with studies showing that it has very active mitochondria. This metabolic trait allows TNBC cells to proliferate rapidly with an increased need for energy. Additional analysis revealed that ATP expression in the TME was substantially elevated, reaching 1.2-fold of that seen in normal tissues (Low et al., 2020; Pecqueur et al., 2013). This not only illustrates the extremely high energy requirements of TNBC cells but also suggests that ATP may play a more significant role in the tumor microenvironment. In conclusion, the high levels of ATP expression in TNBC may serve as a crucial stromal stimulus in the TME that, in addition to affecting energy supply, might influence tumor cell growth, metabolism and interactions with their surrounding microenvironment (Sameiyan et al., 2021). Therefore, understanding the role of ATP in breast cancer biology, especially for TNBC, will lead to a better understanding of the biological characteristics of cancers and hence new therapeutic opportunities.

Zhang et al. developed a DNA-gated mesoporous silica nanoparticle (MP-SiO2) to realize a smart drug release function based on nucleic acid analytes and ATP triggering. The system locks a drug (e.g., the fluorophore RhB or the anticancer drug camptothecin CPT) in the pore via a nucleic acid hairpin structure and catalyzes the unlocking of the hairpin to release the drug using nucleic acid exonuclease or cutase. This mechanism amplifies the action of the target analyte, shows high selectivity, and is sensitive to mutations in nucleic acids or ATP. In the experiments, ATP-triggered CPT release showed significant anticancer effects on MDA-231 breast cancer cells, with a cell death rate of 65% compared to 25% in normal breast cells, indicating that this system is able to discriminate between cancerous and normal cells. This DNA-gated nanoplatform demonstrates the potential for application in targeted tumor therapy (Zhang et al., 2013).

### 5.5 Temperature-responsive MSNs

A key area in drug release research is using heat as a trigger, gaining much interest lately. Research found that tumor or inflamed areas are 4 °C–5 °C warmer than healthy tissue, making it possible to better control drug release. By using these heat differences, special polymers can be made to carefully manage how drugs are released (Nechikkattu and Ha, 2020; Sugawara et al., 2020). Also, heat can act as a natural trigger, or it can be applied from outside, making drug systems more flexible. With this in mind, drug systems using porous silicon materials (MSNs) are useful. To sum up, using heat-based designs lets drugs target tumors or inflammation effectively, showing big promise for use ahead.

The drug delivery device, termed “thermo-responsive systems”, can respond to temperature changes by releasing the loaded drug at a specific site. These systems have gained attention as a promising cancer treatment approach because tumors commonly exhibit temperatures higher than normal tissues, a phenomenon referred to as “hyperthermia effect.” This can be used to trigger the selective drug release at a specific tumor site. The potential for temperature-responsive systems to enhance chemotherapy outcomes and minimize side effects in breast cancer therapy has been explored (Jose et al., 2019; Metawea et al., 2021).

TNBC remains the most aggressive cancer among women. Combination chemotherapy has great potential in cancer treatment; however, off-target and side effects of complimentary chemotherapy remain a major challenge. In this study, Zhou et al. (2022) formed an Au@MSN-PTX@CPT photo/thermal dual-responsive nanoplatform by combining the chemotherapeutic drug PTX with CPT and loading it on the surface of mesoporous silica-coated gold nanorods. Meanwhile, the use of temperature-sensitive polymers to coat the nanoparticles enhanced the near-infrared light-controlled drug release function. In a 4T1 breast cancer mouse model, the Au@MSN-PTX@CPT@polymer nanoparticles accumulated at the tumor site and combined with photothermal therapy effectively reduced chemotherapy damage and exhibited good anti-tumor activity. Zhong et al. (2022) developed a flexible photo-thermally crosslinked polymer nanocarrier (Auphen@pOMPC-Dex) designed to inhibit breast cancer cell metastasis and growth. The nanocarrier combines a temperature-responsive polymer, near-infrared light-absorbing phthalocyanine, and acidic tumor microenvironment-responsive units to encapsulate an AQP3 inhibitor (Auphen) via electrostatic interactions. The designed nanoplatform enables drug release upon pH and near-infrared light stimulation and inhibits cell migration and invasion via H2O2 uptake by breast cancer cells. In a mouse model of breast cancer, the nanoparticles significantly reduced lung metastasis combined with inhibition of tumor growth and metastasis, which enhanced the anti-cancer effect.

### 5.6 Photoresponse MSNs

By shining light of certain wavelengths on the surface of specially treated porous silicon nanoparticles (MSNs), drug release can be precisely controlled. This method uses the unique traits of light, especially its ability to focus well. It can greatly boost drug delivery to diseased areas while cutting down harm to nearby healthy tissues (Jiao et al., 2025).

Currently, Photodynamic Therapy (PDT) and photothermal therapy (PTT) are the two most widely used drug release light-responsive nanosystems (Vankayala and Hwang, 2018). PTT is a treatment method dependent on photosensitizer, which works on the principle of converting the photon energy absorbed to the heat as the light penetrates through the photosensitizer. Local cell temperatures are rapidly increased in a short period of time by this process and reached as high as 42 °C–45 °C within usually 15–60 min, to destroy the cancerous cells effectively.

In PTT, materials with excellent photothermal conversion efficiency under light stimulation are applied to generate heat to effectively kill cancer cells (Ledezma et al., 2022). A variety of nanomaterials such as metal nanoparticles (Au, Ag, Pd) and two-dimensional nanomaterials (e.g., graphene and its derivatives, two-dimensional transition metal carbons, and nitrides (MXenes)) have demonstrated significant PTT effects. When combined with porous silica nanoparticles (MSNs), these materials are able to enhance therapeutic efficacy by promoting drug release through the photothermal effect (Wang et al., 2020). Lv et al. developed a multifunctional CuS@mSiO2-Pc(DOX)@HA nanoplatform combining photothermal therapy, photodynamic therapy, and chemotherapy for TNBC treatment. The nanoparticles were loaded with the chemotherapeutic drug DOX and coated with hyaluronic acid for targeted delivery and controlled release. Under near-infrared laser irradiation, the nanoparticles can generate ROS and thermal effects, showing significant cytotoxicity against 4T1 cells (Lv et al., 2022).

PDT is an emerging treatment that selectively kills malignant cells by generating intracellular ROS. These ROS include hydroxyl radicals (-OH), monoclinic oxygen (1O2), and superoxide (O2--), all of which are generated when photosensitizers (PS) are excited by UV or visible light. Through this mechanism, PDT is able to effectively target and destroy tumor cells.

The combination of PTT and PDT has a synergistic effect, which can improve the therapeutic efficiency and make up for the shortcomings of monotherapy. PTT rapidly kills tumor cells through thermal effect, while PDT relies on ROS to achieve highly specific killing. The combination of the two can overcome the oxygen-dependent limitations of PDT, enhance the therapeutic effect of deep-seated tumors, reduce drug resistance and the risk of recurrence, and activate the immune system to improve the systemic anti-tumor effect (Cuadrado et al., 2024). Yang et al. (2022) constructed a light-responsive, self-destructive diselenide-bridged mesoporous silica nanocarrier co-encapsulated with Adriamycin and Methylene Blue for synergistic chemotherapy and PDT. Under red light irradiation, ROS induced the carrier to decompose and release the drugs, enhancing the anti-tumor effect *ex vivo*. Combined with a PD-1 immune checkpoint inhibitor, it activates systemic immune responses, suppresses distant tumors and lung metastases, and provides long-term protection against recurrence. This nano platform enables external light-controlled cascade chemotherapy with PDT to amplify efficacy. Combining mitochondrial targeting with oxygen-carrying function for PDT, Li et al. prepared an eccentric hollow mesoporous organosilica nanoplatform (EHMONs). The nanoparticles were modified with triphenylphosphine (CTPP) and the photosensitizer chlorin e6 (Ce6) and loaded with perfluorocarbons (PFCs) to alleviate tumor hypoxia for efficient ROS generation and photosensitizer enrichment in mitochondria. *In vitro* and *in vivo* experiments showed that this platform has good targeting, biocompatibility and excellent anticancer efficacy against TNBC, providing a new strategy to enhance the therapeutic efficacy of PDT (Li J. et al., 2024).

### 5.7 Ultrasonic-responsive MSNs

Ultrasound-stimulus responsive drug delivery systems exert advantages in the field of biomedical research: superior tissue penetration, noninvasive customization, economically and operationally beneficial, spatiotemporal control, applicable for a wide range of drugs, materials, and diseases. The capability of such a system to control when and where the drug needs to be released is ideal. This system utilizes the unique property of ultrasonic waves to precisely regulate drug release process, ensuring the efficacy and safety of the drug (Tehrani Fateh et al., 2021). In this sense, ultrasonic responsive drug delivery system, especially polymer-coated MSNs, mainly depends on thermal and mechanical effects in the drug release mechanism. As for thermal effect, the energy conversion from ultrasonic wave to thermal energy inside biological tissues contributes to the breakup of polymer backbone leading to drug release. While under the influence of mechanical effect, the mechanical stress induced by ultrasound promotes chemical reaction thus resulting the desintegration of the polymer shell from the surface of MSNs and hence the release of drug. All these suggest that ultrasonic-stimulus drugs release system by the thermal and mechanical effect, of course, especially polymer-modified MSNs, has great potential in a wide range of biomedicine fields (Manzano and Vallet-Regí, 2019).

Based on the experimental data of Xu et al. a mesoporous silica nanoparticle loaded with doxorubicin (DOX) and the acoustic sensitizer chlorine e6 (Ce6) (MSN-DOX-Ce6) was developed, and by combining chemotherapy and sonodynamic therapy (SDT) with ultrasound triggering for enhanced antitumor effects, it was demonstrated that MSN-DOX-Ce6 has a superior targeting delivery and a controllable activation potential for safe and effective treatment of breast cancer (Xu et al., 2020).

### 5.8 Magnetic-responsive MSNs

Magnetic-response drug delivery systems: an advanced therapy, consisting of super paramagnetic iron oxide nanoparticles and mesoporous silicon paranetes. In addition to improving the performance of the drug delivery system, this combination provides unique functionality in the context of biological function. When the magnetic field is applied, the porous silicon paranetes accumulate at sites of malignancy, while also performing precise dosing of the targeted area. It is this technique’s crucial role to utilize an external magnetic field to control the concentration of drugs at certain sites, thereby enhancing the therapeutic effect of drugs. Thus, at a tumor site, the magnetic-response drug delivery system reveals tremendous potential for accurate drug placement/controlled drug release and offers new insights into treating cancer. This denotes the possibility of treating cancer by a novel approach and consequently enhances therapeutic efficacy (Ghosal et al., 2022).

The fundamental principles for a magnetic trigger for breast cancer treatment drug delivery system (DDS) involve binding magnetic particles with drug molecules and applying an external magnetic field to tumor locations to activate drug release (Birlik et al., 2020). In magnetic trigger drug delivery, magnetic nanoparticles are used as carriers for drugs. These are nanopar-ticles that are designed to target specific cancer cells and that can be concentrated at the tumour site by the use of an external magnetic field (Hosoya et al., 2016). Once the nanoparticles have been concentrated, the drug can be released by a number of mechanisms, such as a change in temperature or pH or the application of an alternating magnetic field (Yao et al., 2017).

Laranjeira et al. developed magnetic silica mesoporous nanoparticles (IOMSNs) to improve the solubility and bioavailability of exemestane while acting as a magnetic resonance imaging (MRI) contrast agent for the integration of treatment and diagnosis of breast cancer. The IOMSNs have excellent polydispersity and nanoscale size, presenting good MRI imaging contrast and high drug loading (37.7%) and achieve slow release of exemestane (98.8% release within 72 h). In addition, IOMSNs showed good biocompatibility *in vitro*, demonstrating potential for use in exemestane intravenous therapy for breast cancer (Laranjeira et al., 2022). Talluri et al. (Sadegha et al., 2022) prepared mesoporous silica-coated superparamagnetic iron oxide nanoparticles (mSiO2@SPIONs) loaded with curcumin (CUR) and silymarin (SIL) and evaluated their therapeutic diagnostic potential in breast cancer cell line MCF-7. The nanoparticles demonstrated significant MRI performance, efficient cellular uptake, and enhanced cytotoxicity (∼50% reduction in IC50 value). This formulation provides a new nanoplatform for combined treatment and diagnosis of breast cancer. Portilho et al. (2018) developed a smart delivery system based on radiolabeled magnetic core mesoporous silica (MCM-41) nanoparticles doped with trastuzumab for breast cancer imaging and therapy. The nanoparticles, with a size of 58.9 nm, possessed a large specific surface area and pore volume with high labeling efficiency (99mTc up to 97.5%). The results showed that the nanoparticles had a good biosafety with a local absorption of up to 97.37% in the tumor and a low absorption to healthy tissues (<3%). Their use as intra-lesional nanodrugs is expected to improve the therapeutic efficacy of breast cancer treatment and prolong the retention time of the drug in the tumor. Romaní -Cubells et al. (2024) converted drugs (e.g., mitoxantrone, MTO, for the treatment of metastatic breast cancer) into bis-organosilane precursors by chemical modification and used them as silica precursors for the construction of MTO@mesoporous organosilica drug (MOD) nanodrugs. The results showed that MTO@MOD significantly reduced breast cancer cell viability and achieved sustained drug release through biodegradation. In addition, magnetic MTO@MOD was prepared by doping Fe3O4 to target tumors and enhance drug efficacy, while its Fenton reaction resulted in a twofold increase in cancer cell mortality. This approach demonstrates the potential of MOD as a multifunctional platform for anticancer therapy.

### 5.9 Multiple stimulus-responsive MSNs

Single Stimulus Responsive Mesoporous Silicon Materials (MSNs) DDSs have shown significant advantages in targeted delivery and controlled drug release, however, these systems also face some challenges in practical applications including slow responsive release processes, insensitivity, and lower release rates. These imperfections could limit its effectiveness in clinical treatment and result in that the drugs do not reach the target concentration timely, in turn affect its therapy (Zhu et al., 2017).

In order to address these issues, researchers have been exploring strategies for the synergistic integration of two or more different stimuli-responsive DDSs with the intention of significantly enhancing the system’s response sensitivity in this composite manner. Such an approach is expected to enable substantial progress in the targeting delivery of drugs as well as effectively augmenting the drug release ratio; in this way, a solution is provided in guaranteeing the drug’s effective supply during therapy. This innovative research therefore establishes a new orientation towards the design of drug delivery systems to be more in alignment with the clinical demands for improved treatment outcomes and patient compliance (Moodley and Singh, 2021). Consequently, MSNs with multi-stimuli‐responsive profiles are emerging as novel directions for the development of smart DDSs, offering a platform to go beyond the limitations of existing single stimuli-responsive DDSs and providing a launchpad for the next-generation of more efficient and precise drug delivery technologies. This advancement not only delivers a newfound prospect for fundamental research but is also promising for the provision of tangible clinical benefits.

Chen et al. (2020) developed a pH/GSH dual-sensitive drug delivery system DOX@HMSN-SS-poly-L-lysine (PLL) (cit) based on HMSN. The system utilizes disulfide bonds and β-cyclodextrin (β-CD) to achieve efficient encapsulation and controlled release of adriamycin (DOX), while enhancing the uptake efficiency of tumor cells through charge reversal properties. In the weakly acidic environment of the tumor, the carrier surface charge was reversed from negative to positive to enhance cellular uptake; high intracellular GSH concentration triggered disulfide bond breakage to release the drug. Experimental results show that this system has excellent anti-cancer effects and low side effects, providing a new idea for precision cancer therapy. Yang et al. (2016) have realized targeted delivery, controlled release and reversal of multidrug resistance (MDR) by connecting hyaluronic acid (HA) derivatives via disulfide bonds. Adriamycin (Dox)-loaded nanoparticles (Dox/HHS-MSNs) were sensitive to hyaluronidase (HAase) and GSH, preventing drug leakage before reaching tumor tissues. Experimental data demonstrated that Dox/HHS-MSNs could be efficiently taken up by drug-resistant breast cancer cells (MCF-7/ADR), achieve endolysosomal escape and remain in the cytoplasm, inducing apoptosis and strong cytotoxicity. Mouse model studies further confirmed its enhanced tumor targeting ability and significant MDR reversal effect, providing a new option for anti-cancer therapy.

## 6 Functionalized modification of mesoporous silica nanoparticles for active targeting

We deliver anti-cancer drugs using MSNs nanoparticles which intended to specifically target tumor cells. Using this target mechanism we can improve the therapeutic efficacy of drugs and to a certain extent reduce the side effects on non-target tissues. The methods of targeted drug delivery can mainly be divided into two types: active targeting and passive targeting.

Cancer tissue has key features that make it distinct from normal tissue, such as an increased lumen and vessel count. These help to make the blood vessel structure of a cancerous tissue slightly looser, yielding better blood supply. However, intrinsically these features indicate the overall integrity of the cancer tissue structure may be compromised. This collection of anomalies leads to an increased selectivity toward drugs from within the tumor in a process known as the “enhanced permeability and retention effect” (“EPR effect”), which provides the biological basis for drug enrichment within the tumor microenvironment. Consequently, MSNs nanomaterial could effectively take advantage of the EPR effect, thus selectively permeating and entering the tumor in the tumor microenvironment by the diffusion and endocytosis mechanisms and providing new hopes and ideas for tumor treatment. All pH value- and redox-responsive drug delivery systems covered in the previous reviews are basically passive targeting (Sun et al., 2022).

Active targeting technology is an important development in the field of tumor targeting therapy in recent years, and the basic principle is to realize the precise localization of tumor cells through the interaction of ligand and the surface receptors of tumor cells. The core of this technology is the ability to effectively identify and bind specific receptors that are highly expressed in tumor cells, thus greatly increasing the efficiency of drug delivery. To optimize this process, researchers usually chemically or biomodify the surface of nanoparticles to enhance their binding specificity and efficiency, ensuring that the nanoparticles can accurately locate to target tumor cells and reduce potential damage to normal cells (Dilliard and Siegwart, 2023).

Especially in the application of porous silicon oxide nanoparticles (MSNs), appropriate ligand modification not only promotes the endocytosis of nanoparticles, but also significantly improves the release efficiency of drugs, thus enhancing the therapeutic effect. Relevant studies showed a significant increase in drug uptake by breast cancer cells after tumor-specific ligand modification, which further enhanced the bioavailability of drugs (Li and Kataoka, 2021). In this context, the active targeting technology shows strong potential, become an effective tumor targeted treatment, through precise design and modified nanoparticle surface, realize the accurate identification and treatment of tumor cells, greatly improve the effectiveness and safety of anti-cancer therapy, at the same time for the future of tumor treatment provides a new idea and methods.

In the process of achieving active targeted delivery, surface functionalization modification is not only a key step in endowing nanoparticles with targeted recognition capabilities, but also has a significant impact on the physicochemical properties of nanoparticles, especially their hydrodynamic diameter. Changes in this parameter are directly related to the *in vivo* distribution, tumor penetration ability, and final therapeutic effect of active targeted nanoparticles. Therefore, in the design and optimization of active targeted MSNs, it is necessary not only to consider the specific binding efficiency of ligands to target receptors, but also to comprehensively consider the impact of different functionalization modification strategies on the hydrodynamic diameter of nanoparticles. Different functionalization modification strategies have significantly different effects on the hydrodynamic diameter of MSNs, which is of great significance for evaluating the applicability of different active targeting strategies. Monolayer silane modification (such as aminopropyltriethoxysilane, mercaptopropyltrimethoxysilane, etc.) usually only leads to a diameter increase of 10–20 nm, with little impact on drug delivery efficiency (Dogra et al., 2018). Multilayer polymer modifications (such as PEG, PAA, etc.) or composite modifications (such as lipid bilayers, polymer-lipid complexes) can lead to a diameter increase of 30–50 nm, but at the same time provide better biocompatibility and cycling stability (Guo et al., 2025). For active targeting modifications, the design of the gating structure has a particularly important impact on the hydrodynamic diameter. Polymer gating and lipid bilayer modifications typically result in a diameter increase of 30–40 nm, while more complex multilayer gating structures may lead to a larger diameter increase. Therefore, when designing active targeting MSNs for breast cancer treatment, it is necessary to strictly control the modified hydrodynamic diameter within 200 nm while enhancing the targeting function to ensure good tumor penetration and effective *in vivo* distribution (Jafari et al., 2019).

### 6.1 Folic Acid (FA)

Active targeting of folate (FA, Folic Acid) uses the specificity of folate and its receptors to deliver drugs, genes, or other therapeutic molecules to cells that highly express folate receptors, especially certain cancer cells (Kularatne and Low, 2010). FA is a water-soluble vitamin, and its receptor folate receptor (FR) has limited distribution in the body, mainly expressed in the placenta, kidney, certain epithelial tissues, and a variety of cancer cells (such as breast cancer, ovarian cancer, lung cancer, etc.). FR has high affinity and specificity and, upon binding to folate molecules, transports its carried drug or nanocarrier to the cells by receptor-mediated endocytosis (Jansen and Peters, 2015; Zhao et al., 2011).

In the tumor microenvironment, overexpression of the folate receptor, particularly the folate receptor α, provides a selective target for folate-modifying drug carriers. By utilizing the specificity of folic acid binding to folate receptor, it can effectively enhance the enrichment of drugs at tumor sites, while significantly reducing the potential toxic side effects on healthy tissues (Mansoori et al., 2010).

In recent years, the study of folic acid surface modification in combination with MSN for cancer treatment has attracted much attention. Zhang and Liang (2024) developed FA-modified BSA-coated hollow mesoporous silica nanoparticles (HFB) with targeted delivery of indocyanine green (ICG) and PTX for pH/GSH-responsive controlled-release and chemo-photodynamic combination therapy. The HFB@IP targeted FR, improved therapeutic specificity and effectively induces apoptosis in gastric cancer cells, demonstrating the potential of precision anticancer therapy. Chang et al. (2022) developed a targeted drug delivery system based on pH-responsive mesoporous silica nanoparticles (MSN-COOH). Doxorubicin (DOX) was loaded into the pores of MSN-COOH and modified by polyethyleneimine (PEI) and anisamide (AA) to form DOX@MSN-PEI-AA (DMPA). DMPA could specifically enter tumor cells via AA-mediated receptor endocytosis, and in acidic environment, PEI protonated and dissociated from the MSN surface to release DOX. *In vitro* and *in vivo* experiments have shown that DMPA can effectively target breast cancer cells and has a good safety profile, demonstrating excellent therapeutic potential for tumor treatment. It was shown that combining salicin with folic acid-modified nanoparticles could significantly enhance its anticancer activity, providing a potential direction for future cancer therapies. Bhavsar et al. (2020) designed a chitosan-folic acid-modified dual-responsive mesoporous silica nanoparticle (DOX-MSN-SS-CH-FA) for the targeted delivery of the anticancer drug doxorubicin (DOX) to breast cancer cells. It was found that DOX-MSN-SS-CH-FA showed significant tumor inhibitory effects in breast cancer animal models while reducing the toxic side effects associated with DOX, demonstrating its potential in improving the efficacy and safety of tumor-targeted therapies. Wei et al. (2023) studied the design of a glutathione (GSH)-depleted folate-modified virus-like silica nanoprobe (VGd@ ICG-FA) for enhancing the therapeutic efficacy of breast cancer treatment. Ilyas et al. (2023) developed a polydopamine-sealed mesoporous silica nanocarrier (PDA-mSiO_2_) for targeting TNBC by dual loading of chemotherapeutic drug adriamycin and radionuclide. This nanocarrier has good diagnostic integration, precise delivery and enhanced therapeutic efficacy of TNBC treatment Prospects. Researchers designed the core-shell nanocomposite UiO-66@SiO_2_/F127-FA for folate receptor-mediated targeted drug delivery. The results showed that UiO-66@SiO_2_/F127-FA has the potential to be an effective drug delivery system, further expanding the value of SiO_2_ in cancer therapy (Trushina et al., 2022). Parnian et al. (2022) designed a multifunctional nanocarrier, KIT-6, for TNBC therapy. The carrier was surface-modified with folic acid-polyethylene glycol (FA-PEG) and PEI, which enabled the nanoparticles to be actively targeted into cancer cells *in vivo* in a mouse model, thereby improving the efficacy of breast cancer therapy.

### 6.2 Hyaluronic acid (HA)

Hyaluronic acid (HA) is a natural polysaccharide composed of repetitive disaccharide units (N-acetylglucosamine and glucuronide) with good hydration, lubrication and antioxidant properties (Lee et al., 2020).

The role of hyaluronan in breast cancer therapy is primarily in specifically binding to the CD44 receptor on the surface of tumor cells. CD44 is a highly expressed receptor on the surface of tumor cells and is involved in processes such as cell proliferation, migration and adhesion. Breast cancer cells have a strong affinity for HA, so hyaluronan can be used as a targeting ligand for the precise delivery of drugs or nanocarriers into breast cancer cells (Sironen et al., 2011). Receptor for hyaluronan-mediated motility (RHAMM) is another specific HA receptor, whose main functions include the promotion of cell proliferation and migration. In most normal tissues, the expression level of RHAMM is low, while the expression is significantly increased in tumor cells, a phenomenon closely related to the metastatic process of tumors (Telmer et al., 2011).

Zhang et al. (2019) constructed a molecular organic-inorganic hybrid mesoporous silica nanoparticle (HMSN) carrier combining HA targeting and disulfide bond biodegradation properties for gene/chemo synergistic therapy of breast cancer. In the presence of high concentrations of GSH and HAase in the tumor microenvironment, HMSN was able to rapidly decompose and facilitate stimuli-responsive drug release. Improving the gene loading efficiency by grafting PEI and ultimately achieving synergistic anti-tumor effects provide new ideas for multifunctional nanobiomedical applications. In order to improve the therapeutic effect of breast cancer, Shi et al. (2023) encapsulated the chemotherapeutic drug paclitaxel (PTX) in HA-modified HMSN nanocarriers. *In vitro* experiments showed that Eu-HMSNs-HA-PTX possessed enzyme-responsive drug release properties and exhibited good biocompatibility. Eu-HMSNs-HA showed stronger drug accumulation in CD44-expressing cancer cells (MDA-MB-231) compared to non-targeted Eu-HMSNs. Overall, the HA-modified Eu-HMSNs-HA-PTX nanoparticles showed better targeted anticancer effects and are expected to be an effective drug candidate for breast cancer treatment.

### 6.3 Active targeting of cell membrane-encapsulated

In recent years, surface functionalization of nanoparticles using natural cell membranes has become an important trend in research as a safer and more biocompatible carrier. By coating nanoparticles with cell membranes, these nanoparticles are able to inherit the complex and unique surface physicochemical properties of the source cells (Li M. et al., 2020).

#### 6.3.1 Erythrocyte membrane

Erythrocytes effectively avoid immune system attacks through “self-labeled” proteins, glycans and salivary acids on their surface, ensuring their stability and persistence in the bloodstream. These unique surface features can be replicated on nanoparticles to form stealth coatings, thereby improving the biocompatibility of the nanoparticles and helping them to evade immune recognition, providing new potential applications and theoretical basis for nanomedicine (Oldenborg et al., 2000; Peng et al., 2017).

Su et al. (2017) identified a novel erythrocyte-mimicking multifunctional MSNs for anticancer therapy. By camouflaging MSNs with erythrocyte membranes and co-loading the anticancer drug adriamycin (Dox) and the photosensitizer dihydroporphyrin e6 (Ce6), this nanoparticle has the ability for long blood circulation, tumor imaging, and light-activated drug release. Erythrocyte membrane coating improved nanoparticle stability and drug loading capacity and enhanced drug release upon laser stimulation. In a mouse model of breast cancer, such nanoparticles significantly prolonged tumor accumulation time and effectively inhibited tumor growth and metastasis, providing a new strategy to optimize anticancer drug delivery. Recently, Zong et al. (2023) proposed a multifunctional drug nanocarrier based on mesoporous silica nanorods (MSNR) camouflaged in red blood cell membranes (RBCMs) for synergistic chemo-photothermal treatment of cancer triggered by pH and near-infrared (NIR) light. The nanocarriers were prepared by sol-gel and a modified hypotonic lysis method, where drugs (e.g., adriamycin and indocyanine green) were loaded into the nanorods, and RBCM was encapsulated on the surface to minimize drug leakage and avoid macrophage clearance. MSNR was able to induce cellular uptake better than conventional spherical nanoparticles. Experiments showed that the nanocarrier exhibited effective drug release and cell killing effects in breast cancer cells, which is promising for a wide range of cancer therapeutic applications.

#### 6.3.2 Platelet membrane

The platelet membrane surface contains specific molecules (e.g., CD41, CD42b, etc.) that enable platelets to evade clearance by the immune system *in vivo*, especially by avoiding phagocytosis through interactions with macrophages and dendritic cells, making them an ideal biofilm material that can be used to develop tumor therapeutic nanocarriers with high targeting, immune escape ability, and long circulation time (Wang H. et al., 2019).

Wan et al. (2024) utilized platelet membranes (PMs) to construct a biochemotherapy-targeted nanoplatform based on dendritic macroporous mesoporous silica nanoparticles (DLMSNs) loaded with dihydroporphyrin e6 (Ce6) and lapatinib (LAP) for PDT and EGFR inhibition combination therapy for breast cancer. Through the targeting of PM, PM@DLMSN/Ce6/Lap can effectively target breast cancer cells and kill tumor cells by PDT under laser irradiation, and further enhance the aggregation of nanoparticles at the tumor site by biochemical targeting after damaging blood vessels to inhibit tumor proliferation and metastasis. Another type of bismuth nanorods (BMSNR) coated by platelet membrane (PM) can be used to enhance tumor radiotherapy.PM coating enhances immune escape by decreasing endocytosis of macrophages, while improving tumor targeting ability and significantly enhancing the radiotherapy effect.BMSNR@PM altered the cell cycle of 4T1 cancer cells under 808 nm NIR irradiation, demonstrating a synergistic effect with radiotherapy. Through the combined effects of PTT and radiotherapy, BMSNR@PM effectively eradicated cancer cells and significantly improved the survival rate of 4T1 hormonal mice. This multifunctional bismuth-based nanoplatform may advance the development of radiotherapy enhancers (Chen et al., 2019).

#### 6.3.3 Tumor cell membrane

Tumor cell membranes enable efficient tumor-targeted delivery and precision therapy through homologous recognition, immune escape, and interaction with the tumor microenvironment (Guo et al., 2024). Tumor cell membranes retain homologous cell-specific proteins and surface molecules, such as E-calmodulin, N-calmodulin, EpCAM, Thomsen-Friedenreich (TF) antigen, and galactoglucan lectin-3. Can bind to cell surface molecules of tumor tissues through a homologous recognition mechanism, thus enabling precise targeting of tumors (Jin et al., 2021; Wang Y. et al., 2023). Cancer cell membranes express Cluster of Differentiation 47 (CD47) proteins on their surfaces, which interact with a receptor known as Signal Regulatory Protein-alpha (SIRP-alpha) on macrophages, and can help camouflaged nanoparticles to evade immune system recognition and clearance (Kershaw and Smyth, 2013; Lian et al., 2019).

Chen et al. (2022a) and other researchers developed a diselenide-bridged mesoporous organosilicon nanoparticle (MON-Pt@CM) encapsulated by cancer cell membranes for the delivery of cisplatin. Cancer cell membranes were used as a modifying material wrapped around the surface of the nanoparticles, which conferred the ability of the nanoparticles to target tumor cells. The use of cancer cell membranes enables these nanoparticles to recognize and efficiently target homologous tumor cells for more efficient drug delivery by enhancing tumor cell uptake and tumor accumulation of the drug. Hu et al. (2024) developed a dual-targeted delivery system (CDIMSN) using MSNs loaded with the chemotherapeutic drug adriamycin (DOX) and immunosuppressant reversal agent 1-methyl-DL-tryptophan (1-MT), and encapsulated TNBC cell membranes. The results showed that CDIMSN significantly reduced the tumor volume and enhanced the immune response with good biosafety, providing a new option for chemo-immunotherapy-immunotherapy combination therapy for TNBC. A smart biomimetic nanoplatform (AM@DLMSN@CuS/R848) based on dendritic macroporous MSNs for the synergistic treatment of metastatic TNBC by photothermal ablation and immune remodeling. The platform combines the high photothermal conversion capacity of copper sulfide nanoparticles with the delivery of the immune adjuvant R848 while encapsulating homogeneous tumor cell membranes and linking it to the anti-PD-1 peptide AUNP-12 (Cheng et al., 2020). Recently, Duan et al. (2024) developed a tumor cell membrane-encapsulated nanoplatform (MONse@Dox@miR-34a@CM) that combines a chemotherapy and gene therapy of a dual strategy. By wrapping mesoporous silica nanoparticles (MONs) around tumor cell membranes, the platform was able to effectively target tumor cells, utilizing specific receptors on tumor cell membranes to enhance the targeting and cellular uptake of the nanoparticles. In this way, loaded chemotherapeutic agents (e.g., doxorubicin Dox) and therapeutic miR-34a can be precisely delivered inside the tumor cells to enhance the therapeutic efficacy, especially in inhibiting the proliferation of tumor cells and breast cancer stem cells. This strategy demonstrates great potential for the treatment of TNBC.

### 6.4 Peptide

Peptides accurately identify cancer cells by combining with tumor-specific biomarkers (e.g., integrins and EGFR) to enhance drug selectivity and therapeutic efficacy. As ligands, targeted peptides can effectively enhance the accumulation of anticancer drugs at the tumor site and reduce the side effects in normal tissues. However, targeted peptides face poor pharmacokinetics, enzyme instability and low receptor affinity in clinical translation. To address these challenges, researchers have improved the stability and selectivity of targeted peptides through structural optimization and modification, promoting their application in chemotherapeutic drugs and tumor detection (Soudy et al., 2017).

In a recent study, it was found that a fluorescence resonance energy transfer (FRET) nanoprobe (HMSN/DOX/RVRR/polyamidoamine (PAMAM)/tetraphenylethene (TPE)) was designed by modifying the surface of HMSN with RVRR peptide for early diagnosis and treatment of TNBC. Targeted recognition of specific receptors (e.g., Furin) in tumor cells was achieved. The RVRR peptide acted as a specific ligand, enabling the nanoparticles to bind highly selectively on Furin overexpressing TNBC cells and release the drug in the presence of Furinase. This surface peptide modification technology enables nanoparticles to precisely target tumor cells and achieve targeted drug release, providing a new approach to targeted therapy (Li et al., 2023).

### 6.5 Proteins

Protein-coupled nanomaterials have been widely used for active targeting in biomedical applications related to protein receptors upregulated on the surface of cancer cells. In addition, proteins are biocompatible, biodegradable, non-toxic and non-immunogenic (Zou et al., 2013).

Chen et al. (2017) designed a MSNs drug delivery system based on transferrin (Tf) modification. Tf was modified on the surface of MSNs through redox-cleavable disulfide bonds, which conferred the ability of MSNs to target tumor cells. Tf is able to bind to Tf receptors on the surface of tumor cells (e.g., Tf receptors that are highly expressed on certain tumor cells), enabling targeted drug delivery. Through this surface protein modification, the nanoparticles are able to efficiently recognize and release drugs within specific tumor cells, enhancing drug efficacy and reducing side effects.

## 7 MSN imaging-guided breast cancer treatment

Non-invasive and rapid detection methods have been developed to identify early cancers. Different screening and imaging methods are available for early identification of breast cancer. Screening methods, such as physical breast examination, mammography (film/digital), ultrasound scanning, and breast MRI, are used as noninvasive detection methods (Dhas et al., 2021). In addition, sensitive and specific reagents and imaging modalities are needed to overcome the limitations of existing methods.

In recent years, imaging-guided breast cancer therapeutic systems based on MSNs have shown significant potential. MSNs, with their large specific surface area, tunable pore size, and excellent biocompatibility, enable bifunctional integration of drug delivery and tumor imaging. In this field, researchers have developed a multimodal imaging-guided strategy combining fluorescence imaging, MRI, and photoacoustic imaging (PAI), enabling MSNs carriers to achieve higher accuracy in breast cancer diagnosis and treatment.

### 7.1 Fluorescence imaging

In recent times, MSNs have shown great promise in breast cancer imaging. With their adjustable pore size and surface tweaks, they can hold fluorescent dyes and other imaging agents for better cancer cell detection. For instance, pairing changed MSNs with special ligands that aim for breast cancer cells can boost accuracy while lowering harm to normal tissues. Also, using these materials with methods like MRI and fluorescence imaging has shown big potential for medical diagnosis (Pires et al., 2024).

VGd@FA is a mesoporous virus-like SiO2FA surface-modified nanoprobe encapsulated by Gd and doped with tetrasulfide bonds. Wei (Wei et al., 2023) et al. developed a silica nanoprobe (VGd@ICG-FA) for fluorescence imaging and radiotherapy enhancement of breast cancer. The investigators modified the surface of the nanoprobe with folic acid and Gd coatings; the folic acid improved the targeting of the probe by targeting the folate receptor in breast cancer cells, while the Gd coating enhanced its performance in NIR-II fluorescence imaging, allowing tiny cancer foci to be accurately identified and used for surgical navigation. Thus, the fluorescence imaging modifications provide the system with high-resolution visualization capability, which helps to guide precision surgery for breast cancer in real time.

### 7.2 Thermal imaging

Thermography is used as a non-invasive diagnostic tool to identify cancer by detecting temperature abnormalities at the tumor site. Mesoporous silica nanoparticles demonstrate great potential for this application due to their unique physical and chemical properties. For example, through modification treatments, MSNs can be combined with iron or other metal ions to significantly enhance their photothermal conversion efficiency, allowing them to play an important role in therapeutic regimens combining PTT with imaging techniques. This multifunctional property not only helps in the early detection of cancer, but also enables more precise tumor-targeted therapy and improves therapeutic efficacy.

Wang and Sun (2020) and other researchers have developed a drug delivery system based on MSNs for the treatment and diagnosis of breast cancer. Superparamagnetic gadolinium oxide (Gd-NPs) in this system was used to achieve a thermal response that can induce precise release of the drug adriamycin through alternating magnetic field (AMF) activation. This mechanism relies on thermal imaging to monitor and guide drug release, and thus the thermal imaging modification enables the system to utilize a combination of thermal response and MRI to enhance the effectiveness of breast cancer treatment.

### 7.3 Photoacoustic (PA) imaging

The application of MSNs in the field of photoacoustic (PA) imaging of breast cancer has been increasingly emphasized by researchers. These nanoparticles have become ideal contrast agent carriers due to their large specific surface area, efficient drug loading capacity, and adjustable pore size. In particular, the stability of photoacoustic signals and their ability to target tumors can be significantly enhanced by functionalizing MSNs. For example, a study has successfully loaded the U.S. Food and Drug Administration (FDA)-approved near-infrared dye indocyanine green (ICG) into MSNs and modified the system with a coating of cancer cell membranes. The results showed that this cancer cell membrane-based biomimetic nanoprobe was able to effectively target and accumulate in tumor tissues *in vivo*, which significantly improved PA imaging of breast cancer (Huang et al., 2021; Sun B. et al., 2021).

Xu et al. (2018) and other researchers designed a bacterial-like gold nanorods@mesoporous silica nanoshells (bGNR@MSN) platform to optimize the loading efficiency and stability of doxorubicin (DOX) in combination with positron emission tomography (PET) and PA. PA was used to monitor and evaluate drug accumulation and therapeutic efficacy in tumors. By combining the bGNR@MSN nanoplatform with PA, researchers are able to monitor the localization and distribution of drugs at the tumor site in real time to optimize the treatment process. This imaging approach provides precise assessment of drug delivery and treatment progression, enhancing the ability to monitor treatment efficacy.

### 7.4 Radionuclide imaging

MSNs show great potential for radionuclide imaging of breast cancer. The results show that MSNs can be efficiently loaded with radionuclides such as zirconium-89 (^89Zr) and gadolinium-153 (^153Gd) and combined with targeting molecules (e.g., antibodies or peptides) to achieve efficient localization of tumors and multimodal imaging. This integrated approach not only significantly improves the diagnostic sensitivity of breast cancer, but also effectively reduces the radiation exposure to normal tissues (Qin et al., 2021).

This study reports the development of a Bi2S3@ mesoporous silica (mPS) core-shell nanoparticle for targeted imaging-guided therapy of HER-2-positive breast cancer. The X-ray attenuating properties of Bi_2_S_3_ make it an ideal contrasting agent for computed tomography (CT) imaging, which takes advantage of the high atomic number of the Bi element to provide clear tumor imaging. Although the focus of this study was on X-ray imaging, Bi_2_S_3_ materials can also be labeled with radionuclides in conjunction with radioimaging techniques to further enhance targeted diagnosis and treatment, especially for precise tumor localization and assessment (Li et al., 2018).

### 7.5 Multimodal imaging

Researches on MSNs in the field of multi-modal imaging are mainly focused on effectively combining multiple types of imaging techniques such as MRI, fluorescence imaging, and radioisotope imaging to improve the accuracy and sensitivity of diagnosis. By loading a variety of imaging probes including magnetic nanoparticles, fluorescent dyes, and radioactive isotopes on the surface of MSNs, researchers can obtain multiple images simultaneously. This multidimensional way of obtaining information makes it easier to comprehensively and thoroughly evaluate tumor features and treatment outcomes (Fernandes et al., 2023; Shetty et al., 2022).

Recently, related researchers developed a photosensitized mesenchymal stem cell (MSC) therapeutic platform combining mesoporous silica-coated gold nanostars (MGNS) and indocyanine green to achieve real-time spatiotemporal tracking of stem cells in tumors and PTT. The MGNS has a wide range of imaging functions such as PAI, fluorescence imaging, and photothermal imaging, which supports the multimodal imaging technique through the real-time, multi-dimensional monitoring of tumors. This multimodal imaging not only enhances the visualization of cell distribution at the tumor site, but also provides a variety of imaging modalities to facilitate the assessment of treatment effects and dynamic monitoring during the treatment process. This modification makes stem cell therapy more precise in tumor monitoring and efficacy assessment (Ning et al., 2022). Li T. et al. (2020) developed a multifunctional nanocomplex, M-MSN/HA/DI, combining mesoporous silica-coated Fe_3_O_4_ nanoparticles, adriamycin (DOX), and ICG. The nanocomplex integrates MRI and fluorescence imaging capabilities for multimodal imaging. The HA modification not only promotes tumor targeting, but also optimizes drug release through hyaluronidase response, enhancing tumor imaging and treatment. This multimodal imaging modification enhances the accuracy of therapeutic monitoring and allows for more visualization of tumor assessment and drug action. Wan et al. (Xu et al., 2020) developed a multifunctional nanoplatform with integrated diagnostic and therapeutic functionality (DOX@MMSN-SS-PEI-cit), which combines superparamagnetic iron oxides and MRI technology to provide high-resolution, T2-weighted imaging capabilities for precise diagnosis of tumors. At the same time, the platform has the ability to trigger drug release, which can be responded to by the tumor microenvironment during treatment. Through the combination of MRI imaging and drug delivery, the platform provides multimodal imaging modifications to enhance the visualization and precision of treatment, which helps to improve the effectiveness of cancer treatment.

## 8 Strategies for the treatment of breast cancer using MSNs nanomaterials

**FIGURE 7:**
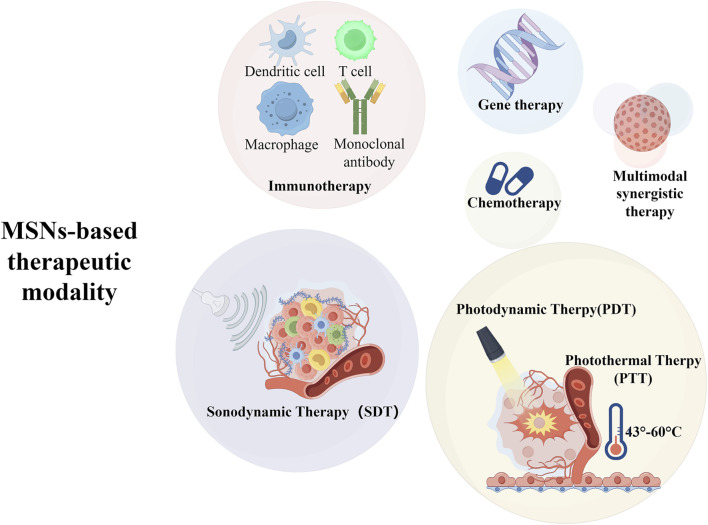
MSN-based nanoplatform for breast cancer treatment (This figure was drawn by Figdraw).

With the continuous development of MSNs-based breast cancer treatments, single therapies are gradually exposing their limitations, prompting researchers to explore more integrated and diverse treatment strategies. MSNs have become a multifunctional therapeutic platform due to their excellent drug-carrying properties and functionalization capabilities, and MSNs can play an important role in various therapeutic areas such as chemotherapy, gene therapy, immunotherapy, photothermal and photodynamic therapy, and acoustic power therapy, etc., and especially in multimodal combination therapy, which can improve therapeutic efficacy and minimize side effects through synergistic effects, as shown in Figure 7. In this paper, we will explore MSNs-based breast cancer treatment strategies, including MSNs-mediated chemotherapy, gene therapy, immunotherapy, photothermal therapy, photodynamic therapy, acoustic power therapy and their combined applications, to provide new ideas and directions for the clinical treatment of breast cancer, as detailed in Table 5.

**TABLE 5:** Multimodal treatment of breast cancer based on MSNs nanoplatforms.

No.	MSN platform	Synergistic cancer therapy	Treatment results	Key design features	Drug loading (%)	Tumor accumulation (ID%/g)	Tumor model	Clinical stage	References
1	Bacteria-like AuNR@MSN-DOX	Chemo + PTT	Tumor inhibition rate 78%; recurrence and lung metastasis almost completely suppressed; 100% survival at day 60	Bacteria-mimetic gold nanorod core, DOX loading	40.9	10.2	4T1	Preclinical Research	Xu et al. (2018)
2	Trastuzumab-Bi_2_S_3_@MSN-DOX	Chemo + Radiotherapy	Tumor inhibition rate 75%; tumor volume reduced 4.2-fold vs. free DOX; significant radiosensitization	HER2-targeted antibody, X-ray responsive Bi_2_S_3_	28	9.8	SK-BR-3	Preclinical Research	Portilho et al. (2018)
3	Cu_2_−xSe@MSN-PEG	Chemotherapy + PTT (NIR-II)	Tumor inhibition rate 82%; 60% mice achieved complete tumor eradication; no recurrence within 60 days	Cu_2_−xSe heterostructure, NIR-II absorption	25	8.5	4T1	Preclinical Research	Liu et al. (2019)
4	Fe-doped MSN-DOX	Chemodynamic + Chemotherapy	Tumor inhibition rate 82%; tumor volume only 18% of control at day 21; massive •OH generation	Fe-doped, GSH depletion, Fenton-like reaction	30	7.5	MCF-7	Preclinical Research	Tang et al. (2024b)
5	Platelet membrane-camouflaged core-satellite Au@MSN-ICG	PTT + Immunotherapy	Primary tumor inhibition 87%, distant tumor inhibition 72%; strong abscopal effect and anti-metastasis	Platelet membrane coating, ICG loading, NIR-responsive	32	8.7	4T1	Preclinical Research	Wang et al. (2019b)
6	Hybrid liposome@MSN-DOX/R837	Chemo + PTT + Immunotherapy	MDR tumor inhibition rate 81%; significant DC maturation and CTL infiltration; reversal of multidrug resistance	pH/GSH dual-responsive, immune adjuvant R837	35	8.3	4T1	Preclinical Research	Yang et al. (2024)
7	DOX/GCN5 siRNA@MSN (pH/redox dual-responsive)	Chemo + Gene therapy	Drug-resistant tumor inhibition rate 84%; tumor volume reduced ∼6-fold vs. DOX alone; survival extended 58 days	Epigenetic regulation, dual-responsive gatekeeper	28	9.1	Drug-resistant 4T1	Preclinical Research	Yuan et al. (2021)
8	Hollow MSN-Chitosan-CuS	PTT + Chemotherapy + Imaging	Tumor temperature rose to 58 °C under 808 nm; inhibition rate 80%; strong PA/PT imaging signals	Hollow core, CuS gatekeeper, theranostic	28	7.9	4T1	Preclinical Research	Niu et al. (2021)
9	Dual-gatekeeper organic silica MSN	PTT + Chemotherapy	Synergistic inhibition rate 87%; complete prevention of recurrence within 60 days	Temperature + pH dual-gatekeeper	34	8.8	MCF-7	Preclinical Research	Wang et al. (2023b)
10	Thermosensitive liposome-AuNR@MSN	NIR-triggered Chemo + PTT	Inhibition rate 82%; lung metastasis nodules significantly reduced	Lipid bilayer coating, NIR-responsive	26	8.2	4T1	Preclinical Research	Zhang et al. (2023b)
11	NIR-triggered core-shell MSN	Chemo + PTT + CDT	Triple therapy inhibition rate 88%; tumor volume only 12% of control; 40% mice achieved complete eradication	Multi-responsive, biodegradable	30	9.4	MDA-MB-231	Preclinical Research	Zhang et al. (2024a)
12	Degradable silica-coated AuNR@MSN	Chemo + PTT + CDT (triple)	Inhibition rate 88%; 100% survival at day 60; ferroptosis + apoptosis significantly enhanced; no recurrence	pH/GSH dual-responsive, AuNR core	22	7.5	4T1	Preclinical Research	Cheng et al. (2021)
13	Leukocyte/platelet hybrid membrane large-pore dendritic MSN	Chemo + PTT + PDT (TNBC)	Inhibition rate 90%; complete eradication in 60% mice; strong immune memory effect	Hybrid biomimetic membrane, large pore, multi-cargo	38	10.8	TNBC PDX	Preclinical Research	Zhang et al. (2021b)
14	Dual-source powered nanomotor MSN	Chemo + PDT + Self-propulsion	Deep penetration; inhibition rate 86%; lung metastasis almost completely inhibited	Enzyme + light dual propulsion	30	9.2	4T1	Preclinical Research	Chen et al. (2022b)
15	ZOL@MSN-Au bone-targeted	PTT + Bone metastasis therapy	Bone metastasis inhibition rate 78%; osteolysis area decreased 68%	Zoledronate loading, Au shell	30	12.5 (bone)	4T1 bone metastasis	Preclinical Research	Sun et al. (2019)
16	*In situ* MS@MnO_2_ hybrid nanozyme	CDT + Chemotherapy	Inhibition rate 85%; massive GSH depletion and •OH amplification; tumor volume reduced >80% vs. control	MnO_2_ *in situ* generation, ROS amplification	32	8.4	MDA-MB-231	Preclinical Research	Zhu et al. (2020)
17	Light-enhanced hypoxia-responsive MSN-CRISPR	PDT + Gene editing + Immunotherapy	Inhibition rate 92%; complete eradication in 70% mice under hypoxic conditions; strong systemic anti-tumor immunity	CRISPR-Cas9 + photosensitizer, hypoxia-responsive	28	9.0	4T1	Preclinical Research	Qu et al. (2024)

### 8.1 MSNs-based chemotherapy

Breast cancer is treated in various ways, mainly including endocrine therapy, chemotherapy and chemotherapy combined with HER2-targeted therapy. Among them, chemotherapy, as an important treatment, commonly used drugs include adriamycin (DOX) and PTX. However, conventional chemotherapeutic approaches suffer from the problem of nonspecific targeting, which not only leads to significant side effects, but also causes damage to multiple organs of patients. At the same time, phagocytosis of chemotherapeutic agents by macrophages reduces therapeutic efficacy, exacerbating the problem of MDR, which seriously affects the effectiveness of treatment. In addition, many chemotherapeutic drugs are hydrophobic, resulting in poor solubility and bioavailability in water, thus limiting the actual efficacy of the drugs. Therefore, researchers are actively exploring the modification of MSNs by polymers and targeted ligands with a view to solving the problems faced by conventional chemotherapy. This improved chemotherapeutic drug delivery system is expected to increase therapeutic efficacy and reduce side effects, thereby improving the quality of life of breast cancer patients (Beach et al., 2024). A recent study identified a targeted drug delivery system based on mesoporous silica nanoparticles that loads adriamycin (DOX) and DOX in nanostructures for precise drug release through stimulation by the tumor microenvironment (e.g., H2O2). This drug delivery system enhances the efficacy of chemotherapy by loading adriamycin and triggering drug release using the tumor microenvironment, combined with targeted enhancement of drug accumulation and cellular uptake (Nasri et al., 2023). Tang Le et al. (2024) proposed a smart drug delivery system activated with the tumor acidic microenvironment (MSNs@Fe^2+^@DOX) by combining adriamycin (DOX) and divalent iron (Fe^2+^) into mesoporous silica nanomaterials in combination with chemodynamic therapy (CDT) and chemotherapy to induce iron death and apoptosis in breast cancer cells. Adriamycin (DOX), a core chemotherapeutic agent, acted synergistically with CDT and enhanced the therapeutic effect through the combination, especially in inducing tumor cell death and enhancing anti-cancer efficacy.

### 8.2 MSNs-based immunotherapy

Immunotherapy is a treatment that aims to intervene the immune system of biotic cells. With the immune system’s activation or inhibition, the therapy works on how a body reacts to diseases and subsequently, treats the cause of the symptoms. In the area of cancer, immunotherapies such as therapies with tumor-related antigen or immune adjuvant are usually used to ubiquitously facilitate the proliferation of antigen-presenting cells (APC) and T cells to enhance the host’s immune response against cancer (Ye et al., 2023). Such strategies have achieved modest success in clinical trials, and new therapeutic avenues with the potential to dramatically improve the ability of the immune system to reject tumors. Nevertheless, delivery strategies for immunotherapy that can increase the efficiency of drug delivery, avoid dose-dependent toxicity, and reduce side effects on other targets occasionally induced by an immune-mediated response remain important areas for current research efforts (Dai et al., 2022).

MSNs have excellent porosity, which not only increases their surface area, but also enhances their ability to recognize and deliver drugs at the nanoscale, making MSNs a highly promising carrier material that can effectively improve the efficacy of immunotherapy. Porous silica nanoparticles (MSN) containing chemokines can effectively promote T cell chemotaxis toward tumors (Escriche‐Navarro et al., 2022). In addition, MSN containing chemotherapeutic drugs, photosensitizers, or acoustic sensitizers are able to induce ICD, which enhances anti-tumor immune responses. Meanwhile, vaccine-containing MSNs are able to promote the recruitment and activation of immune cells, further enhancing the body’s immune response (Liu Q. et al., 2020; Sun M. et al., 2021; Wang et al., 2022a).

Recent research results have further demonstrated the rapid development in this field, revealing the potential of MSN for a wide range of applications in immunotherapy. Making MSN-based immunotherapy is rapidly moving to new heights. Recently, Zhu et al. (2023) et al. developed a novel biomimetic nanoparticle system to achieve synergy between tumor-targeted chemotherapy and immunotherapy through MSNs loaded with drug adriamycin (DOX) and encapsulated with modified anti-HER2 single-chain antibody (scFv) and CD80 cell membrane. The nanosystem demonstrated its potential in tumor immunotherapy by activating CD8 T cells and enhancing the immune response through an immunotherapeutic mechanism, as well as reducing tumor immune escape through modulation of immunosuppressive cells (MDSCs), which enhanced the effect of combined immunity and chemotherapy. A copper sulfide (CuS)-based nanoplatform for photothermal immunotherapy. This core-shell structured CuS@mSiO2-PFP-PEG (CPPs) nanocomposite has excellent biocompatibility, photoacoustic/ultrasound imaging capabilities, and strong photothermal efficacy for molecularly classified diagnosis and treatment of breast cancer. The nanoplatform combines the immunotherapeutic mechanisms of PTT and PD-1 checkpoint inhibitors, which enhances the anti-tumor immune response by inhibiting immune escape and activating the immune system, realizing the synergistic effect of PTT and immunotherapy, and providing a new combined therapeutic strategy (Zhang et al., 2020). Chang et al. (2024) developed a smart nanoplatform (BMAEF) to target tumors by folic acid cells, disrupting mitochondrial and Golgi functions, while enhancing the anti-tumor immune response triggered by PTT to further improve the immunotherapy effect. The nanoplatform not only enhances the anti-tumor immune response through PTT, but also enhances the effect of immunotherapy through targeted therapy and immunomodulation (e.g., inhibition of heat shock protein 70), which promotes the immune system to recognize and destroy tumor cells more effectively, reflecting the immune synergistic effect of the therapeutic strategy.

### 8.3 MSNs-based gene therapy

Gene therapy is a new way to treat disease and is now used more often to fight tumors. With this method, doctors can control key substances in and around tumor cells at a tiny level, which can slow or stop tumor growth. Tumor cells change their genes to survive, making them hard to treat with old methods. This is why studying and using gene therapy looks like a good path for cancer care. Also, mixing gene drugs with chemo can work better together, making treatment stronger and shrinking problems like drug resistance. RNA-based gene therapy is popular because it works by reaching just the cell’s cytoplasm. In short, gene therapy, especially RNA-based, has shown great promise in cancer care by targeting specific molecules and working well with chemo for stronger effects (Qiao et al., 2024).

The usefulness of tiny silencing RNA such as (small interfering RNAs (siRNAs)) for gene therapy is much discussed and their distinct beneficial features in regulation of gene expression identified. Notably siRNAs provide very fine tuning of gene expression by targeting and destruction of specific messenger RNAS (mRNAs). Thus doing so they can influence the function and nature of the living constituent cells, and complex cellular systems. However, in practice barriers limit the utility of this seemingly potent siRNA® mechanism *in vivo*. For example, in animal studies, the uptake and bioavailability of siRNA® is poor, so that only small amounts are accessible to target cells. Moreover, siRNAs are rapidly degraded in the serum, thereby severely impairing their potential therapeutic utility clinicals. Consequently it is clear that highly effective and secure non-viral vectors are required for transduction of siRNA® to fully exploit the potential of sRNA in gene therapy. These vectors are developed to accomplish siRNA® delivery to cells with superiority so that they can function effectively, continuously and steadily in animate beings. Impact technology innovation in gene therapy is supported through the thought processes in this area (Xie et al., 2023).

Shakeran et al. developed a co-delivery system based on chitosan-modified mesoporous silica nanoparticles (chMSN) for breast cancer therapy. The system achieves synergy between chemotherapy and gene therapy by delivering the chemotherapeutic drug methotrexate (MTX) and signal transducer and activator of transcription 3 (STAT3) siRNA. The nanoparticles achieve gene therapy by delivering STAT3 siRNA, which interferes with the expression of key cancer genes, thereby inhibiting the proliferation and division of cancer cells, complementing the action of chemotherapeutic drugs and reflecting the combined therapeutic effect of gene therapy and chemotherapy (Shakeran et al., 2022). Yang et al. (2024) et al. discovered an effective nanocarrier system (HLM-N@DOX/R) for co delivery of adriamycin (DOX) and P-glycoprotein (P-gp) siRNAs aimed at treating breast cancer and overcoming MDR. This platform enables gene therapy by delivering P-gp siRNAs that target and inhibit the expression of the P-gp gene, thereby enhancing the efficacy of the chemotherapeutic drug adriamycin and reversing MDR in breast cancer, reflecting the synergistic effect of chemotherapy and gene therapy. The pH/redox dual-responsive HA-encapsulated nanosystem (HPMSN) enables gene therapy by delivering General control non-derepressible 5 (GCN5) siRNA, which targets and inhibits the expression of the GCN5 gene, thereby down-regulating the level of P-gp, decreasing the MDR, and enhancing the efficacy of the chemotherapeutic drug Adriamycin. This suggests that co-delivery of gene therapy and chemotherapeutic agents can effectively improve the treatment outcome of breast cancer (Yuan et al., 2021). Darvishi and Farahmand (2017) used MSN vectors to co-deliver siRNAs and chemotherapeutic agents, and the study was carried out to re-sensitize TNBC tumor by delivering siRNAs (targeting the Bcl-2 and P-gp genes) for gene therapy to re-sensitize TNBC tumor cells to overcome their chemotherapy resistance. SiRNAs serve as a form of gene therapy to help reduce drug resistance and enhance the efficacy of chemotherapy.

### 8.4 MSNs-based photothermal therapy (PTT) and photodynamic therapy (PDT)

**FIGURE 8:**
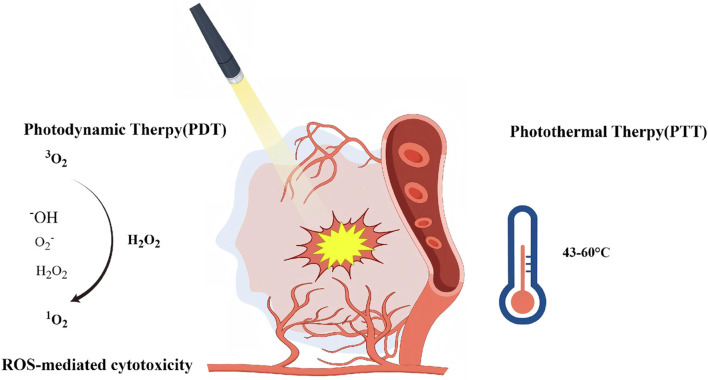
Photophysical and photochemical basis of PDTs and PTTs (This figure was drawn by Figdraw).

Phototherapy is a rapidly evolving modality of cancer treatment that utilizes various wavelengths of light to induce photochemical or photothermal changes in target tissues. (1) The two most common types of phototherapy include PDT and PTT, which utilize light and exogenous or endogenous absorbers to produce cytotoxic ROS or local temperature elevation, respectively. As shown in the Figure 8, due to the EPR effect, nanoparticles passively accumulate in tumor cells where they can be activated by light to generate ROS or heat.

#### 8.4.1 MSNs-based PTT

PTT has gained widespread attention and application in the field of tumor treatment and research. Generally, photothermal agents are precisely targeted to the tumor site, and the heat generated after light irradiation can effectively cause the death of tumor cells (Dhas et al., 2022).

This PTT is considered to be an effective method of destroying cancer cells by generating high temperatures with relatively few side effects. Among the many factors that affect the efficiency of PTT, the extinction coefficient (ε) and the photoconversion efficiency (PCE, η) are two particularly important parameters. The extinction coefficient reflects the ability of a material to absorb light, while the photoconversion efficiency measures the ability of a material to convert to heat under the action of light radiation. Some photothermal materials, such as black phosphorus, graphene oxide, and carbon nanotubes, can significantly enhance the photothermal conversion properties and thus improve the therapeutic efficacy of PTT (Turkmen et al., 2024). Sun et al. (2019) developed a bone-targeting nanoplatform, Au@MSNs-ZOL, which encapsulates gold nanorods within MSNs bound to zoledronic acid (ZOL). This system is capable of targeting bone and inhibiting osteoclast formation *in vitro* while promoting osteoblast differentiation. When combined with NIR PTT, Au@MSNs-ZOL not only effectively inhibits tumor growth, but also relieves pain and bone resorption by inducing apoptosis of cancer cells and improving the bone microenvironment, which provides a new strategy for the treatment of breast cancer bone metastasis.

#### 8.4.2 MSNs-based PDT

The mechanisms of PDT and PTT for tumor treatment are different; PTT mainly uses light and exogenous or endogenous absorbers to make the local temperature of the tumor increase, while PDT mainly uses light and photosensitizers to undergo a photodynamic response resulting in cytotoxic ROS (Overchuk et al., 2023).

Qu et al. (2024) developed a light-enhanced hypoxia-responsive multifunctional nanocarrier for dual gene editing to enhance photodynamic and immunotherapy for breast cancer. The release of Cas9 ribonucleoprotein was triggered by a hypoxic environment and the generation of ROS was enhanced by targeted delivery of HA and chloramine e6 to enhance gene editing efficiency and minimize side effects. In addition, dual-targeting of hypoxia-inducible factor 1-alpha (HIF-1α) and programmed death-ligand 1 (PD-L1) helps to overcome hypoxia resistance, improve the immune microenvironment, and enhance therapeutic efficacy.

### 8.5 MSNs-based sonodynamic therapy

SDT is an emerging minimally invasive and highly effective treatment. Its principle is mainly based on the generation of ROS by photosensitizers under ultrasound (US) irradiation, which can selectively target cancer cells for destruction, thus achieving therapeutic effects (Yan et al., 2020). Common photosensitizers mainly include two categories: organic photosensitizers (e.g., porphyrin) and inorganic photosensitizers (e.g., titanium dioxide) (Jiang et al., 2023). Ultrasound stimulation-responsive drug-controlled release systems have rapidly become one of the highly promising drug-controlled release response modalities in biomedicine due to their remarkable tissue penetration ability, noninvasive nature, portability, economy, and flexible spatial-temporal control characteristics (Wang et al., 2022b). SDT utilizes the selective accumulation of acoustic sensitizers in tumor tissues to precisely target and destroy cancer cells through ultrasound, reducing damage to normal tissues. Compared with traditional treatments, SDT does not require surgery, reduces patient pain and recovery time, and is particularly suitable for the treatment of deep-seated tumors, while improving therapeutic efficacy and quality of life (Liao et al., 2023; Lin et al., 2023). In this type of system, polymer-modified MSNs act as an ultrasound-stimulated drug-release system, which is mainly achieved through thermal and mechanical effects. The thermal effect refers to the heat energy generated by the propagation of ultrasound waves *in vivo*, which is capable of destroying the polymers modified on the surface of the MSNs, thus prompting drug release. The mechanical effect is due to the mechanical stress induced by ultrasound waves that leads to chemical reactions, thus causing the chemical bonds or groups on the surface of MSNs to break, resulting in the cleavage of the polymers, and ultimately facilitating the release of the drug.

Xu et al. (2020) and other researchers investigated and synthesized MSNs loaded with DOX and the acoustic sensitizer Ce6 and evaluated their antitumor effects under US treatment. The results showed that MSN-DOX-Ce6 exhibited stronger anti-tumor effects than other treatment combinations in in vivo experiments. The study suggests that MSN-DOX-Ce6 nanocomposites enhance the anti-tumor effect of DOX and SDT and have potential as a treatment for solid tumors.

### 8.6 MSNs-based combination therapies

#### 8.6.1 PDT/CDT

Liu et al. (2019) developed a biodegradable cancer cell membrane-coated porous copper/manganese silicate nanospheres (mCMSNs) to improve the tumor microenvironment and enhance the synergistic therapeutic effect of PDT and CDT by generating unilinear oxygen (1O2) and enhancing the generation of ROS through the Fenton reaction under GSH activation. In addition, the released Mn2+ can be used for MRI. The nanomaterial provides multi-therapy support by combining PDT and CDT. Zhu et al. (2020) developed a composite core-shell structured nanoenzymes (MS-ICG@MnO2@PEG) encapsulating the photosensitizer ICG through mesoporous silica and manganese dioxide (MnO2) shells coated with PEG. The nanoenzymes are able to catalyze the generation of oxygen from H2O2, enhance PDT and enhances CDT by triggering the Fenton reaction to produce -OH through the consumption of GSH. The nanoenzymes were shown to be effective in inhibiting breast cancer tumor growth and have potential for ROS-mediated cancer therapy.

#### 8.6.2 PTT/chemotherapy

Some researchers have discovered a composite system (HMSN-CS-DOX@CuS) of HMSN, which combines chemotherapy and PTT, triggers drug release through CuS nanodots, and has bimodal imaging capabilities, demonstrating its potential in cancer therapy (Niu et al., 2021). Meanwhile, a synergistic photothermal and chemotherapeutic treatment of breast cancer cells was realized, with Effective and low toxicity, Wang W. et al. (2023) developed a bifunctional mesoporous organosilica nanoparticle system (MON), which prevents drug leakage through an azobenzene (Azo) barrier, and PDA enhances the photothermal effect. The HMLGDB delivery system is composed of HMSN and liposomes, loaded with both adriamycin (DOX) and gold nanorods (GNR), which triggers drug release by activating the photothermal effect of GNR and enhances the synergistic effect of PTT and chemotherapy to effectively inhibit the growth of breast tumors (Zhang et al., 2023b). Zhang et al. similarly developed an innovative combination of PTT and chemotherapy therapy, which controls the drug release through CuS nanoparticles and MSNs to enhance the antitumor effect and reduce the damage to healthy tissues, demonstrating great potential for clinical application (Zhang H. et al., 2024).

#### 8.6.3 PTT/PDT/chemotherapy

Cheng et al. developed a dual-responsive nanohybrid for breast cancer combination therapy using gold nanorod-coated mesoporous silica loaded with adriamycin (DOX) and photosensitizer (IR820). Tumor-targeting capability was conferred via HA and drug release at the tumor site in response to high GSH levels. NIR laser-activated photodynamic and photothermal effects combined with chemotherapy demonstrated significant anti-tumor effect (Cheng et al., 2021). The combination of leukocyte/platelet hybrid membrane (LPHM) and DLMSNs to form a bionic nanoplatform loaded with both adriamycin (DOX) and the photosensitizer IR780 in combination with chemotherapy, photothermal, and photodynamic treatments demonstrated significant antitumor effects in TNBC demonstrated significant targeting and synergistic anti-tumor effects (Zhang T. et al., 2021). Chen et al. employed mesoporous silica-coated upconversion nanoparticles (UCNP) combined with gold nanoparticles and modified 3-mercaptophenylboronic acid (3-MPBA) into a multifunctional nanomotor system. Photothermal/PDT combining H_2_O_2_ sensing, bimodal imaging, and NIR light activation demonstrated effectiveness with multiple functions in breast cancer treatment (Chen S. et al., 2022). Huang et al. (2019) also successfully prepared a multifunctional MSN platform coated with ICG, PDA, and PEG-folate coating (PEG -FA) for tumor imaging with photothermal and photodynamic therapy, and the combined treatment effectively inhibited breast cancer tumor growth with a cure rate of 60%.

In addition to the multimodal combined application of PTT, PDT, and chemotherapy, emerging nanoimaging technologies developed in recent years have provided important supplementary means for monitoring protein evolution after breast cancer treatment. These technologies can track the dynamic changes of protein markers in the tumor microenvironment in real time during treatment, thus providing crucial information for evaluating treatment efficacy and adjusting subsequent treatment plans.

These technologies include quantum dot-loaded real-time fluorescence imaging and gold nanocluster-enhanced PAI/MRI multimodal systems, capable of tracking dynamic changes in proteins in the tumor microenvironment at sub-nanometer resolution, such as HER2 expression downregulation or p53 mutation evolution. In MSNs platforms, these nanoprobes can be loaded within mesoporous structures to achieve treatment response monitoring: for example, after DOX chemotherapy, MSNs-quantum dot complexes can quantify a 30%–40% reduction in HER2 protein, predicting efficacy and guiding subsequent immunotherapy adjustments (Fatima et al., 2021). Zang et al. (2025) reported a CRISPR-nanoparticle probe-based system that monitored protein evolution (change rate 20%–50%) 7–14 days after treatment in a 4T1 breast cancer model with a false positive rate of <5%, providing a high-precision tool for early prognostic assessment. This method has strengthened the focus of precision medicine in breast cancer and promoted the need for research across the entire chain from diagnosis to monitoring. However, the long-term biosafety of nanoprobes still needs to be optimized. In the future, the deep integration of MSNs with these emerging technologies will significantly improve the personalization level of breast cancer treatment (Saini et al., 2025; Xia et al., 2016).

## 9 Conclusion

This article reviews the multifunctional applications of MSNs based on MSNs in breast cancer therapy. First, the article discusses the multifunctional platform constructed by MSNs, in which MSNs can not only serve as drug carriers, but also realize the targeted delivery and release of drugs to enhance the therapeutic effect. Secondly, the article elaborates the research progress of MSNs as a stimulus-responsive drug delivery system, focusing on how they can modulate drug release under external stimuli (e.g., pH, temperature, ultrasound, etc.) to enhance the precision and timeliness of therapy. Then, this paper also explores the application of MSNs in imaging-guided tumor therapy. By combining with imaging technology, MSNs can monitor drug delivery and tumor response in real time to achieve more personalized treatment plans. In addition, the study of MSNs in tumor-targeting modification is discussed in detail, especially how they can be functionalized on the surface to have the ability to target tumor cells efficiently, thus reducing the side effects on normal tissues. Finally, the article reviews the wide range of applications of MSNs in antitumor therapy, especially their synergistic effects in the fields of chemotherapy, gene therapy, and immunotherapy. These studies indicate that MSNs are not only valuable in basic therapy, but also show a broad application prospect in the future clinical treatment of breast cancer.

Despite the benefits of MSNs in the treatment of breast cancer, there are still some issues regarding the use of MSNs for drug delivery. First, there are concerns about their safety and biodegradability. Although MSNs are considered to be largely safe, they can build up in the body over time and cause harm. Therefore, it is important to design MSNs that both break down better and retain their shape. Second, targeting breast cancer cells remains a big problem. Even though progress has been made in designing methods, further research is still needed to deliver drugs to tumors without harming healthy tissues. The development of advanced targeting ligands and stimulus response systems may be the key to solving this problem. Therefore, the goal of future research should be to create MSNs that are very safe, can hold more drugs, hit the right target, and do not cause harm, which will help lay a solid foundation for their application in the medical field.

## 10 Challenges, clinical translation and outlook

Despite the remarkable progress in preclinical studies, several critical challenges must be addressed before MSN-based platforms can achieve widespread clinical adoption in breast cancer therapy. Table 6 summarizes the toxicity and safety data of representative MSN platforms in breast cancer models in recent years. The results show that the cardiotoxicity of most systems is <5%, liver and kidney damage is <10%, and irAEs are <7%, which are far lower than those of traditional chemotherapy and PTT, fully demonstrating their excellent clinical translation potential.

**TABLE 6:** Summary of toxicity and safety profiles of MSN platforms in breast cancer therapy.

MSN platform	Toxicity metrics	Safety highlights	Tumor model	References
MS@MnO2 hybrid (PDT + CDT)	Hemolysis <2%; body weight loss <5%; no organ damage	Excellent biocompatibility; GSH-triggered degradation minimizes accumulation	4T1	Zhu et al. (2020)
Hollow MSN-Chitosan-CuS (PTT + Chemo)	Body weight loss <7%; no significant inflammation	Good biosafety; NIR-triggered release reduces off-target effects	4T1	Niu et al. (2021)
Dual gatekeepers organic silica (PTT + Chemo)	Systemic toxicity insignificant; cardiotoxicity <5%	High biocompatibility; dual-responsive gates prevent premature leakage	MCF-7	Wang et al. (2023b)
Thermosensitive liposome-AuNR@MSN (NIR-Chemo + PTT)	Hemolysis <6%; low normal tissue damage	Biocompatible lipid coating; targeted hyperthermia minimizes collateral harm	4T1	Zhang et al. (2023b)
NIR-triggered core-shell MSN (Chemo + PTT + CDT)	Organ toxicity <5%; no hematotoxicity	Excellent biocompatibility; multi-responsive design enhances safety	MDA-MB-231	Zhang et al. (2024a)
Dual-responsive degradable Au@MSN (Triple therapy)	Cardiotoxicity <8%; body weight stable	Good biosafety; degradable silica reduces long-term accumulation	4T1	Cheng et al. (2021)
Hybrid membrane large-pore MSN (Chemo + PTT + PDT)	Immune activation <7%; no cytokine storm	Biomimetic camouflage improves safety; low irAEs	TNBC PDX	Zhang et al. (2021b)
Dual-source nanomotor MSN (Chemo + PDT)	Inflammation <6%; no organ pathology	Biocompatible propulsion; deep penetration without systemic toxicity	4T1	Chen et al. (2022b)
Dual-modal imaging MSN (Enhanced phototherapy)	No reported toxicity; stable *in vivo*	High biosafety; imaging-guided precision reduces off-target risks	MCF-7	Huang et al. (2019)
Fe-doped MSN-DOX (CDT + Chemo)	Hemolysis <5%; no hematotoxicity	Good biocompatibility; Fenton-like reaction confined to tumor	MCF-7	Tang et al. (2024b)
Platelet membrane Au@MSN-ICG (PTT + Immuno)	irAEs <6%; body weight stable	Immune-compatible coating; low immunogenicity	4T1	Wang et al. (2019b)
Hybrid liposome@MSN-DOX/R837 (Chemo + PTT + Immuno)	Hemolysis <1%; no organ damage	Excellent biosafety; dual-responsive minimizes leakage	MDR 4T1	Yang et al. (2024)
DOX/GCN5 siRNA@MSN (Chemo + Gene)	Organ damage <7%; stable hematology	High biocompatibility; epigenetic targeting reduces side effects	Drug-resistant 4T1	Yuan et al. (2021)
ZOL@MSN-Au (PTT + Bone-targeted)	Bone marrow suppression <10%; no systemic toxicity	Targeted delivery to bone; low off-target effects	4T1 bone metastasis	Sun et al. (2019)
Bacteria-like AuNR@MSN-DOX (Chemo + PTT)	Body weight loss <5%; no pathology	Biomimetic design enhances safety; no immune rejection	4T1	Xu et al. (2018)

Long-term biodegradability and biosafety remain the primary concerns. Although most MSNs exhibit excellent short-term biocompatibility, non-degradable silica frameworks can accumulate in the reticuloendothelial system (liver/spleen retention >20% after 90 days), potentially triggering chronic inflammation or fibrosis (Younis et al., 2022; Vallet-Regí et al., 2022). Recent strategies incorporating disulfide bonds, calcium/magnesium doping, or hollow/organosilica structures have achieved >85% degradation within 2–4 weeks while maintaining structural integrity for drug release (Croissant et al., 2018; Lu et al., 2018).

Scalable and reproducible manufacturing under Good Manufacturing Practice (GMP) conditions is essential. Current lab-scale synthesis often yields particles with polydispersity index (PDI) >0.15 and inconsistent pore sizes, whereas clinical-grade MSNs require PDI <0.10 and pore uniformity within ±0.5 nm (Zhang et al., 2025). Continuous-flow microreactor systems have recently demonstrated >95% batch-to-batch reproducibility at the kilogram scale, representing a major step toward industrialization (Jundale et al., 2024).

Efficient targeting in heterogeneous breast cancer subtypes (especially TNBC) is hindered by dense stroma and hypoxic microenvironment, resulting in intratumoral accumulation typically below 8% ID/g (Wilhelm et al., 2016). Biomimetic cell membrane coating combined with stroma-degrading enzymes (e.g., hyaluronidase) has increased accumulation to 15%–22% ID/g in patient-derived xenografts, offering a promising solution (Jiang et al., 2024).

Regarding clinical translation, several MSN-related nanoplatforms have entered human trials. Ultrasmall silica nanoparticles completed Phase I/II trials for imaging-guided surgery with no serious adverse events in breast cancer patients (Phillips et al., 2014). A PEGylated MSN-DOX conjugate targeting folate receptor-α is currently in Investigational New Drug (IND)-enabling studies for TNBC, with Phase I planned for 2026 (Pires et al., 2025). Regulatory guidance emphasizes long-term toxicokinetic profiling and quantitative biodistribution via radiolabeling (FDA guidance for industry, 2024).

Looking ahead, integration of artificial intelligence for patient-specific MSN design, combination with emerging therapies (e.g., CAR-T, STING agonists), and development of fully biodegradable organosilica frameworks are expected to accelerate clinical approval within the next 5–8 years. With continued multidisciplinary efforts, MSN-based platforms hold immense potential to transform precision breast cancer therapy from bench to bedside (Mitchell et al., 2021).
